# Publisher Correction: A general and flexible method for signal extraction from single-cell RNA-seq data

**DOI:** 10.1038/s41467-019-08614-2

**Published:** 2019-02-04

**Authors:** Davide Risso, Fanny Perraudeau, Svetlana Gribkova, Sandrine Dudoit, Jean-Philippe Vert

**Affiliations:** 1 Division of Biostatistics and Epidemiology, Department of Healthcare Policy and Research, Weill Cornell Medicine, New York, NY 10065 USA; 2 Division of Biostatistics, School of Public Health, University of California, Berkeley, CA 94720 USA; 3 Laboratoire de Probabilités et Modèles Aléatoires, Université Paris Diderot, 75005 Paris, France; 4 Department of Statistics, University of California, Berkeley, CA 94720 USA; 5 CBIO-Centre for Computational Biology, MINES ParisTech, PSL Research University, 75006 Paris, France; 6 Institut Curie, 75005 Paris, France; 7 INSERM U900, 75005 Paris, France; 8 Department of Mathematics and Applications, Ecole Normale Supérieure, 75005 Paris, France

Correction to: *Nature Communications* 10.1038/s41467-017-02554-5, published online 18 Jan 2018.

The original PDF version of this Article contained errors in two equations. In equation 1, all Γ symbols were inadvertently omitted. In the second equation in the subsection entitled ‘1. Dispersion optimization’ within the Methods section ‘ZINB-WaVE estimation procedure’, all Ψ symbols were inadvertently omitted. These errors have been corrected in the PDF version of the Article; the HTML version was correct from the time of publication.

