# Supplementary material for: A general and flexible method for signal extraction from single-cell RNA-seq data
Source: Nat Commun. 2018 Jan 18;9:284. doi: 10.1038/s41467-017-02554-5 (PMC5773593; doi:10.1038/s41467-017-02554-5)
Supplement: Supplementary file 1 — Supplementary Information [file 41467_2017_2554_MOESM1_ESM.pdf]

Supplementary figures

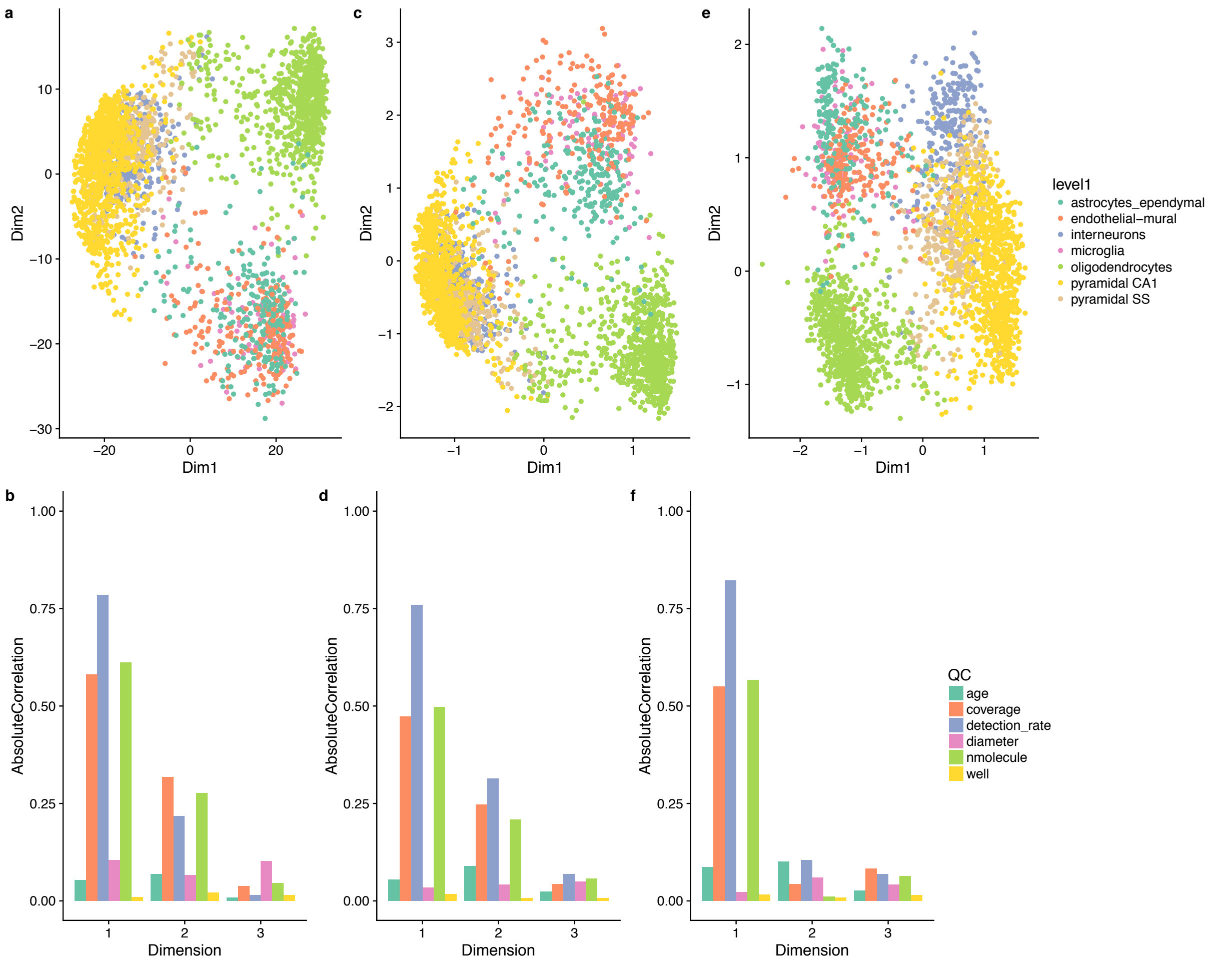

Supplementary Figure 1: *Low-dimensional representation of the S1/CA1 dataset.* Upper panels provide two-dimensional representations of the data. Lower panels provide barplots of the absolute correlation between the first three components and a set of QC measures (see Methods). **(a, b)** PCA (on TC-normalized counts); **(c, d)** ZIFA (on TC-normalized counts); **(e, f)** ZINB-WaVE (no normalization needed). ZINB-WaVE leads to a low-dimensional representation that is less influenced by technical variation and to tighter, biologically meaningful clusters.

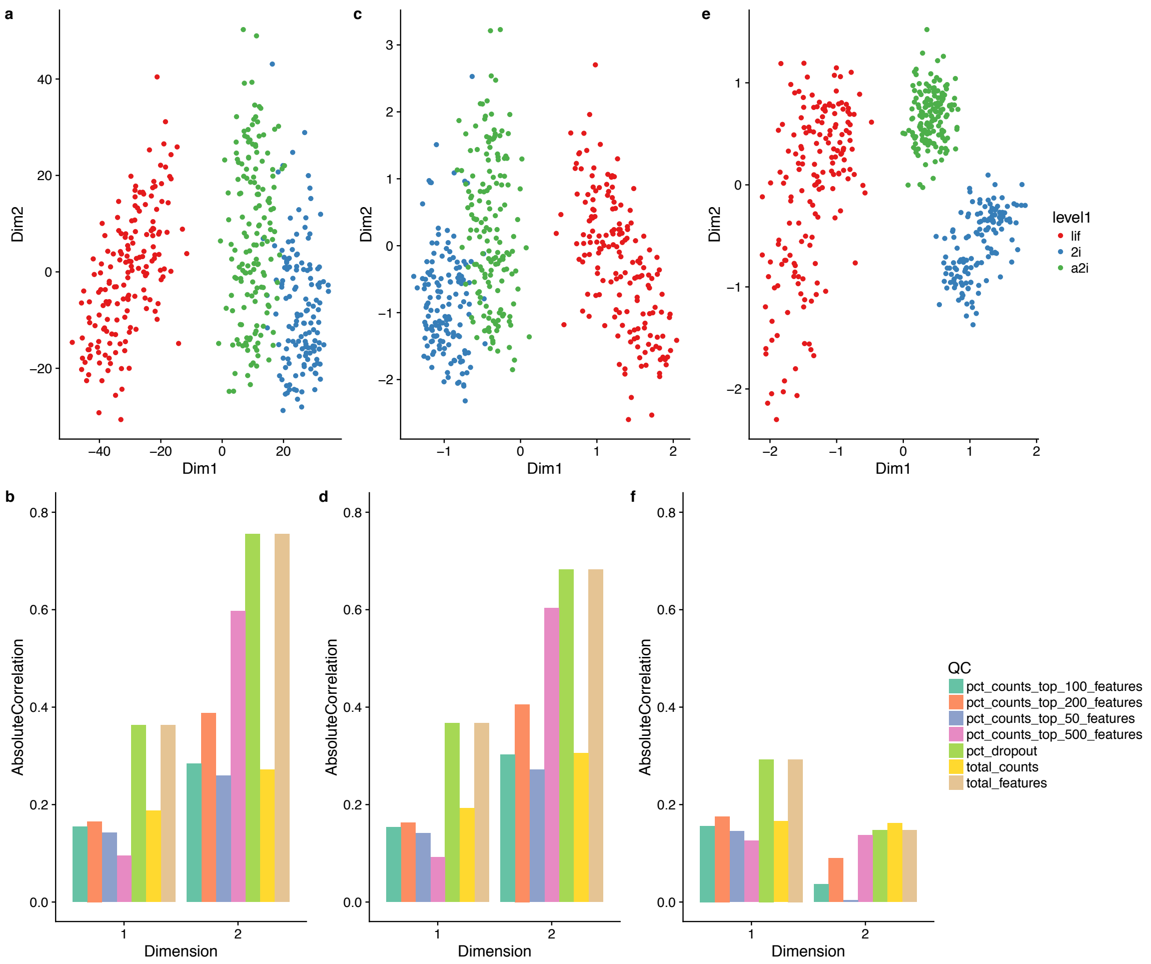

Supplementary Figure 2: *Low-dimensional representation of the mESC dataset.* Upper panels provide two-dimensional representations of the data, after selecting the 1,000 most variable genes. Lower panels provide barplots of the absolute correlation between the first two components and a set of QC measures (see Methods). **(a, b)** PCA (on TC-normalized counts); **(c, d)** ZIFA (on TC-normalized counts); **(e, f)** ZINB-WaVE (no normalization needed). ZINB-WaVE leads to a low-dimensional representation that is less influenced by technical variation and to tighter, biologically meaningful clusters.

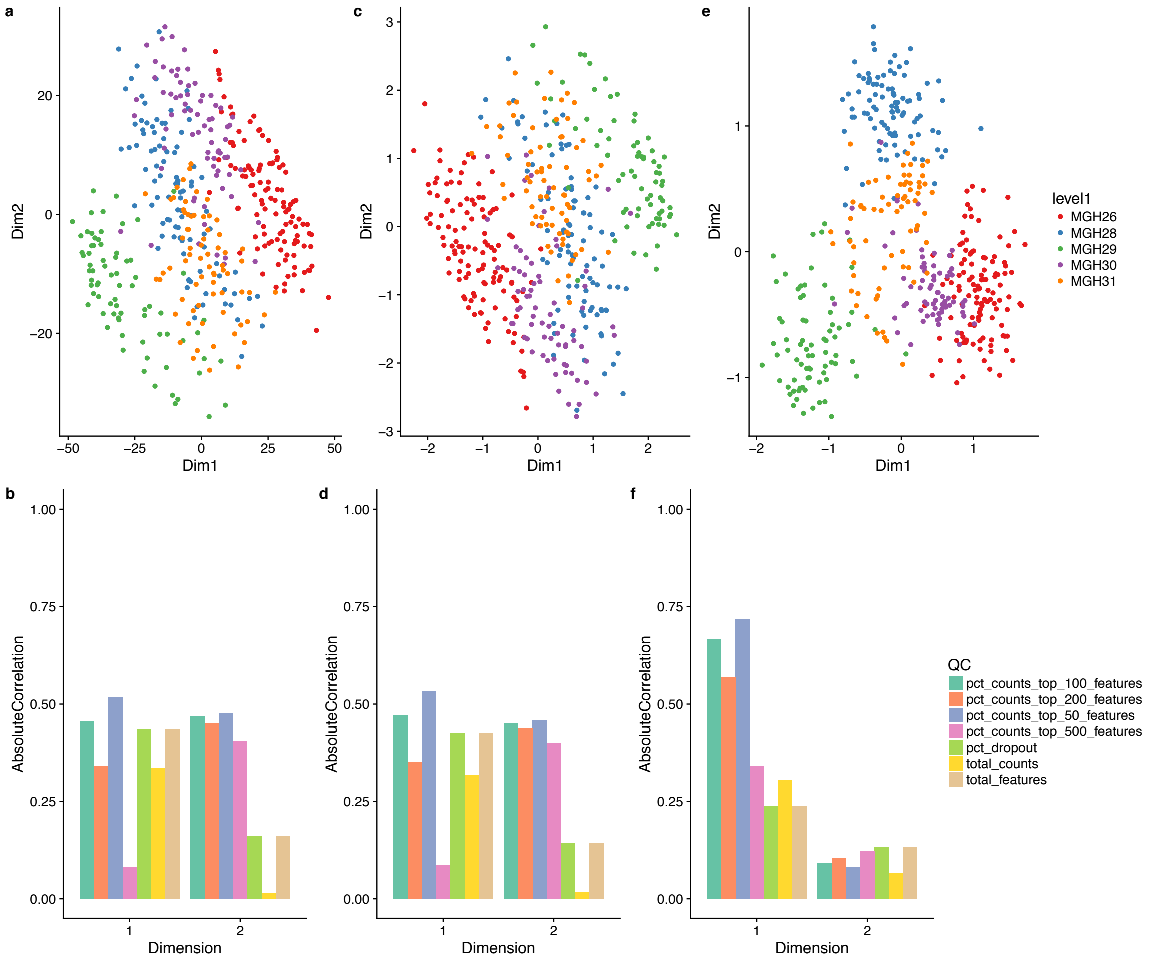

Supplementary Figure 3: *Low-dimensional representation of the Glioblastoma dataset.* Upper panels provide two-dimensional representations of the data, after selecting the 1,000 most variable genes. Lower panels provide barplots of the absolute correlation between the first two components and a set of QC measures (see Methods). **(a, b)** PCA (on TC-normalized counts); **(c, d)** ZIFA (on TC-normalized counts); **(e, f)** ZINB-WaVE (no normalization needed). ZINB-WaVE leads to a low-dimensional representation that is less influenced by technical variation and to tighter, biologically meaningful clusters.

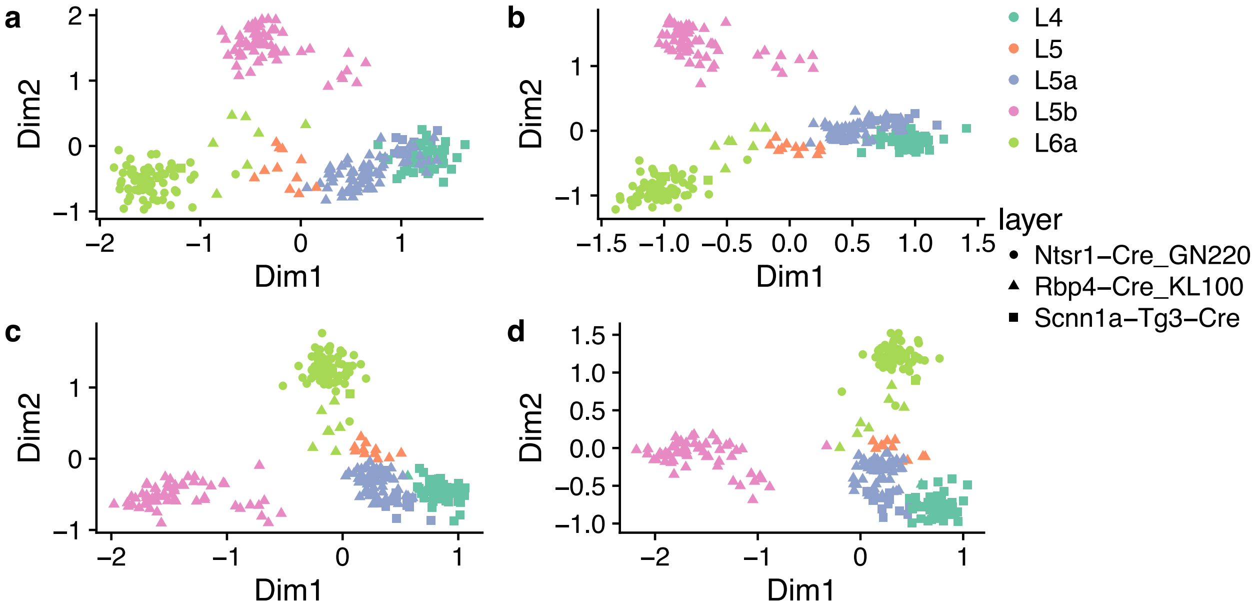

Supplementary Figure 4: *Low-dimensional representation of the V1 dataset.* ZINB-WaVE two-dimensional representation of the data, after selecting the **(a)** 500, **(b)** 2,000, **(c)** 5,000, **(d)** 10,000 most variable genes. ZINB-WaVE leads to a stable low-dimensional representation, robust to the number of highly variable genes selected.

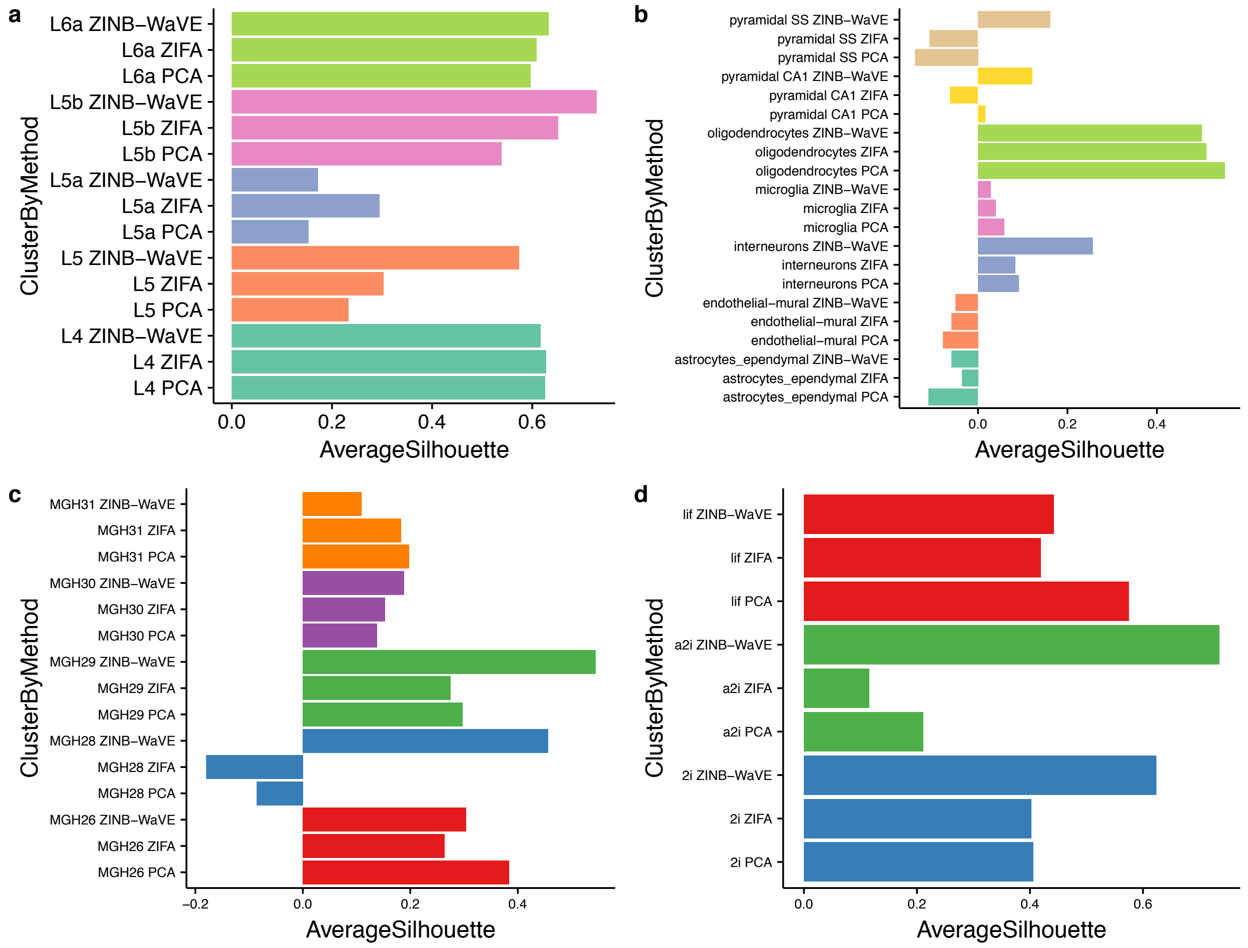

Supplementary Figure 5: *Per-cluster average silhouette widths: Real datasets.* **(a)** V1 dataset; **(b)** S1/CA1 dataset; **(c)** Glioblastoma dataset; **(d)** mESC dataset. For each of the four scRNA-seq datasets of Figure 2 and Supplementary Figures 1 – 3, barplots of the per-cluster average silhouette widths for ZINB-WaVE, ZIFA, and PCA (the best normalization method was used for ZIFA and PCA). Silhouette widths were computed in the low-dimensional space, using the groupings provided by the authors of the original publications: unsupervised clustering procedure **(a–b)**, observed characteristics of the samples, such as patient **(c)** and culture condition **(d)** .

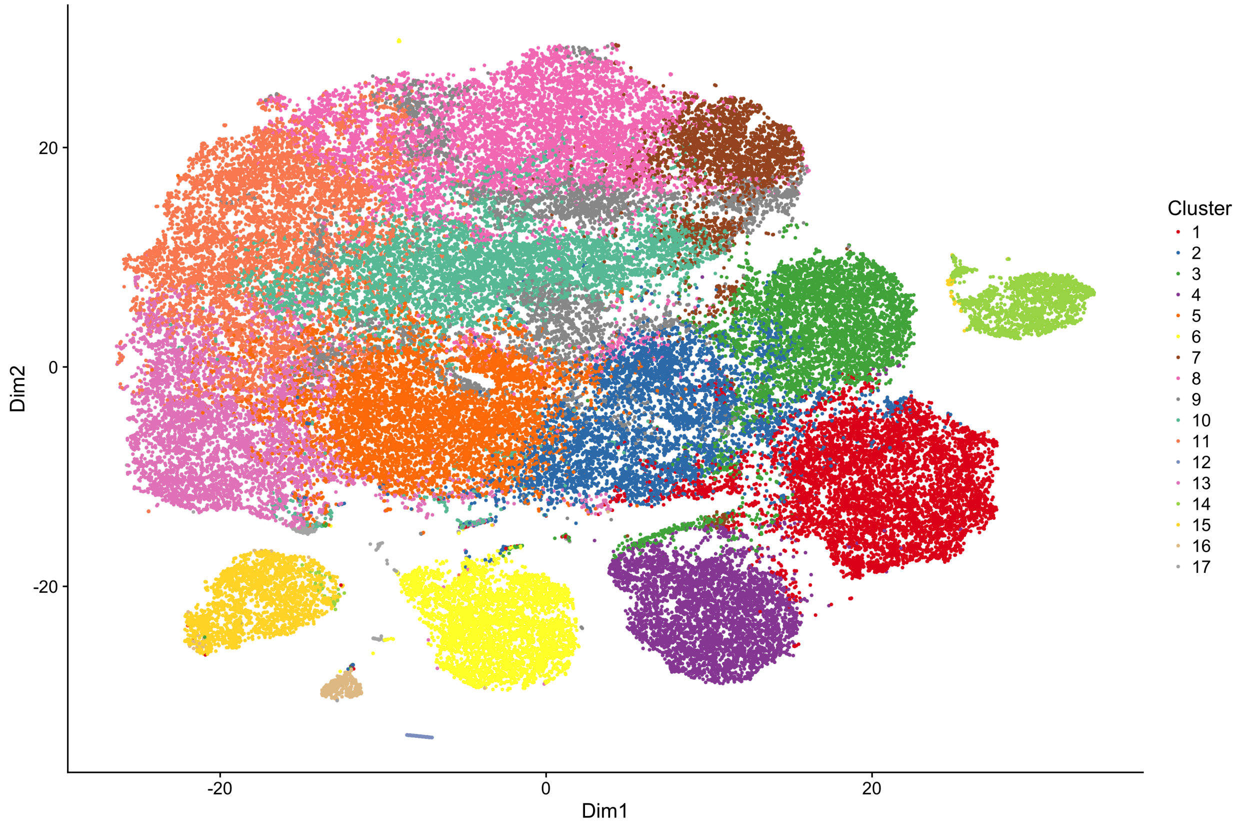

Supplementary Figure 6: *Analysis of the 10x Genomics 68k PBMCs dataset.* Two-dimensional t-SNE representation of W ($K = 10$) color-coded by sequential k-means clustering (see Methods for details on the clustering procedure).

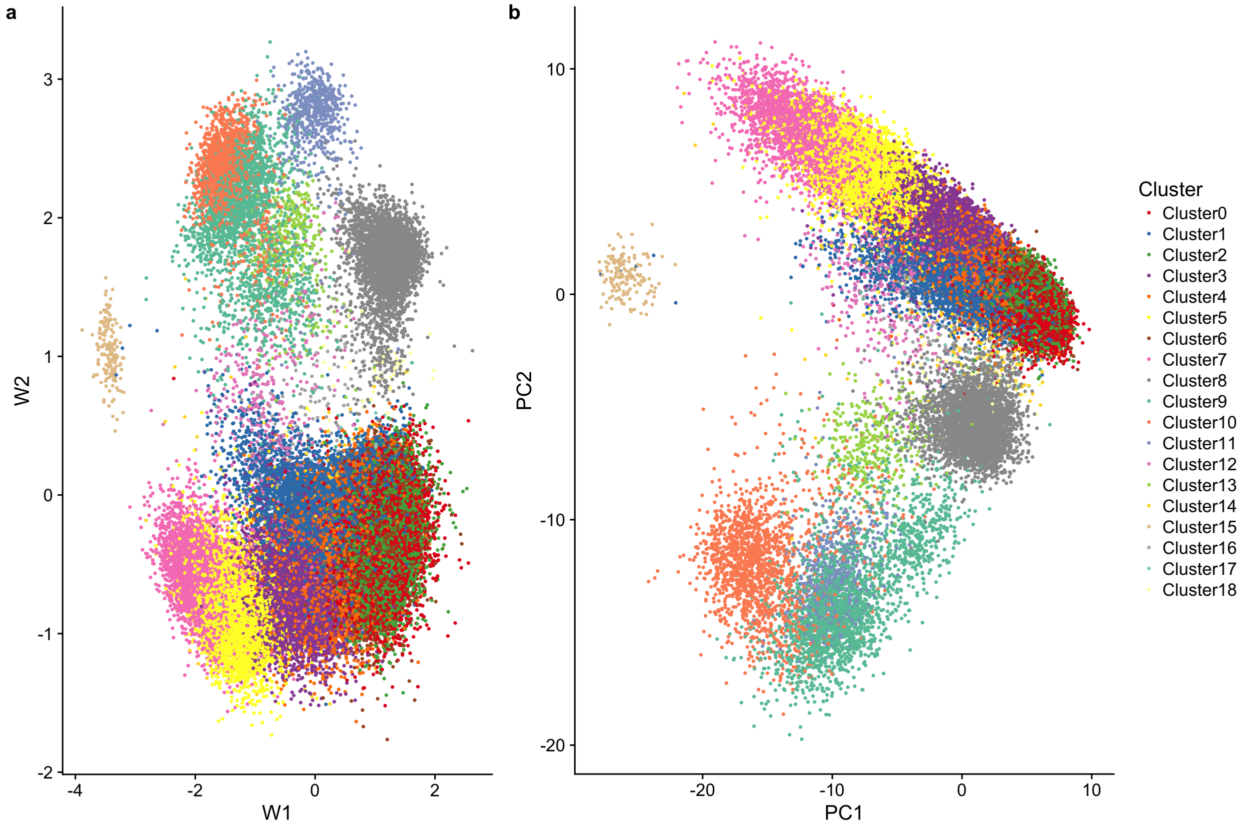

Supplementary Figure 7: *Analysis of the 10x Genomics 68k PBMCs dataset.* **(a)** Two-dimensional signal inferred using ZINB-WaVE. **(b)** First two principal components. Cells are color-coded by sequential k-means clustering (see Methods for details on the clustering procedure).

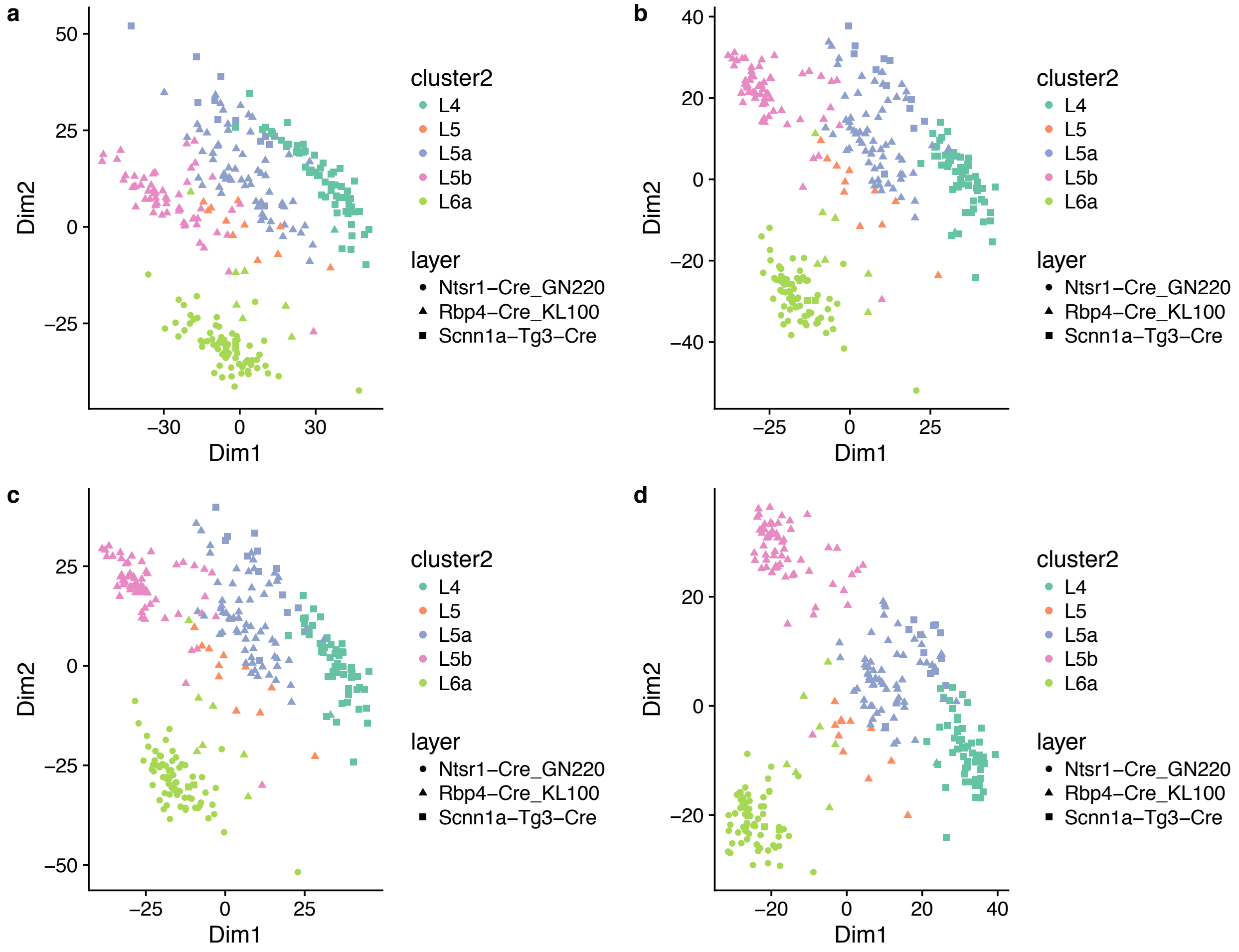

Supplementary Figure 8: *Principal component analysis for V1 dataset.* **(a)** No normalization; **(b)** TC normalization; **(c)** TMM normalization; **(d)** FQ normalization.

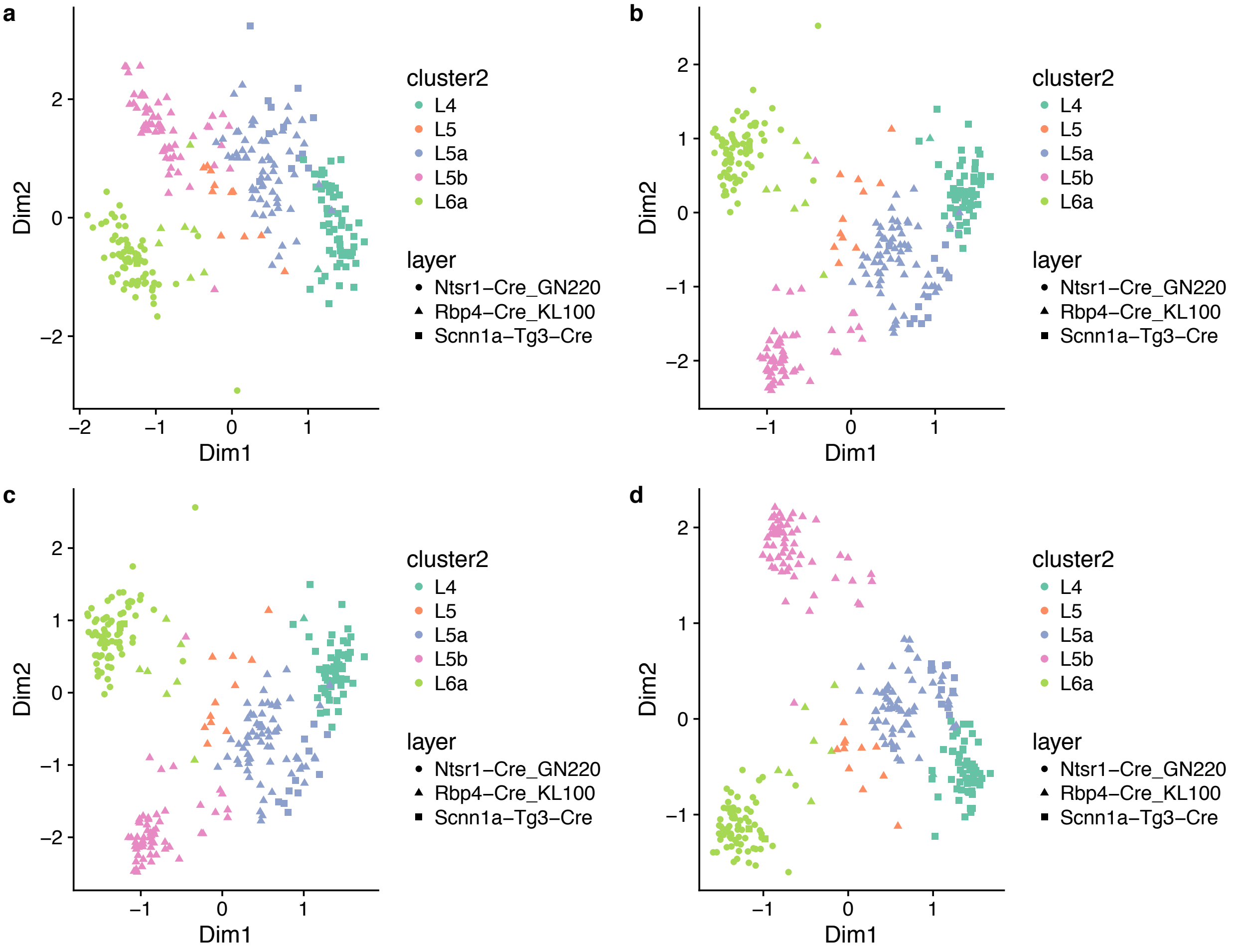

Supplementary Figure 9: *Zero-inflated factor analysis for V1 dataset.* **(a)** No normalization; **(b)** TC normalization; **(c)** TMM normalization; **(d)** FQ normalization.

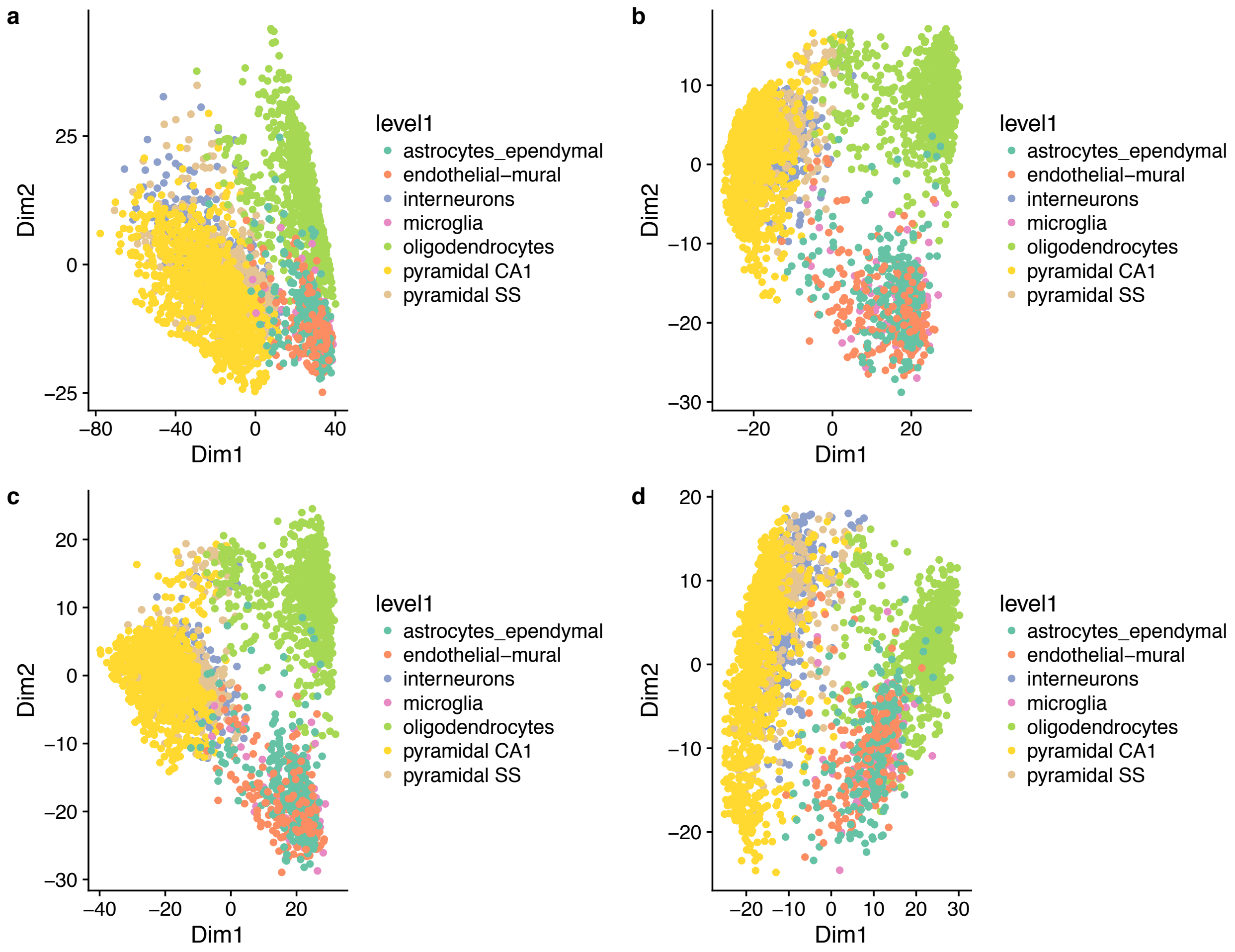

Supplementary Figure 10: *Principal component analysis for S1/CA1 dataset.* **(a)** No normalization; **(b)** TC normalization; **(c)** TMM normalization; **(d)** FQ normalization.

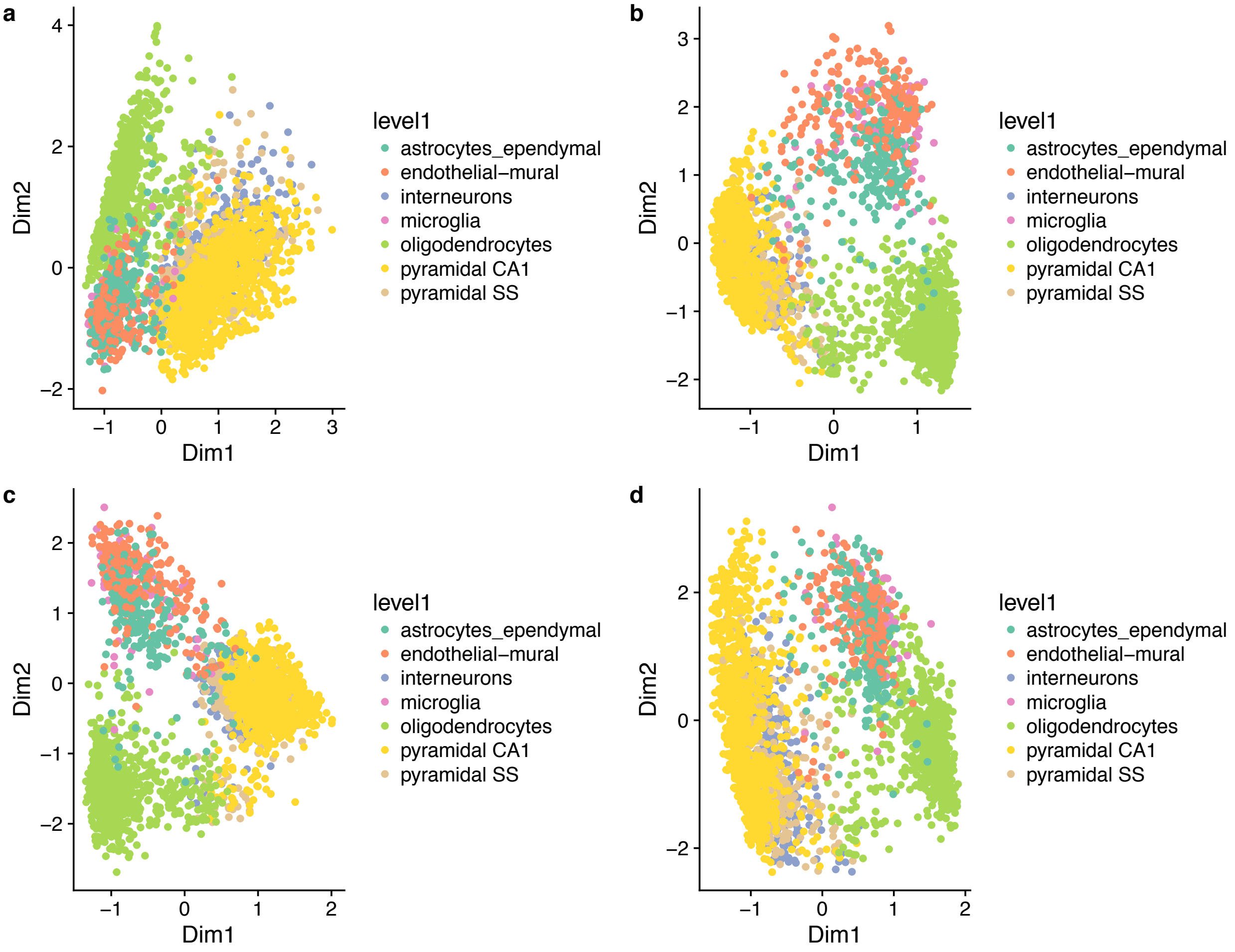

Supplementary Figure 11: *Zero-inflated factor analysis for S1/CA1 dataset.* **(a)** No normalization; **(b)** TC normalization; **(c)** TMM normalization; **(d)** FQ normalization.

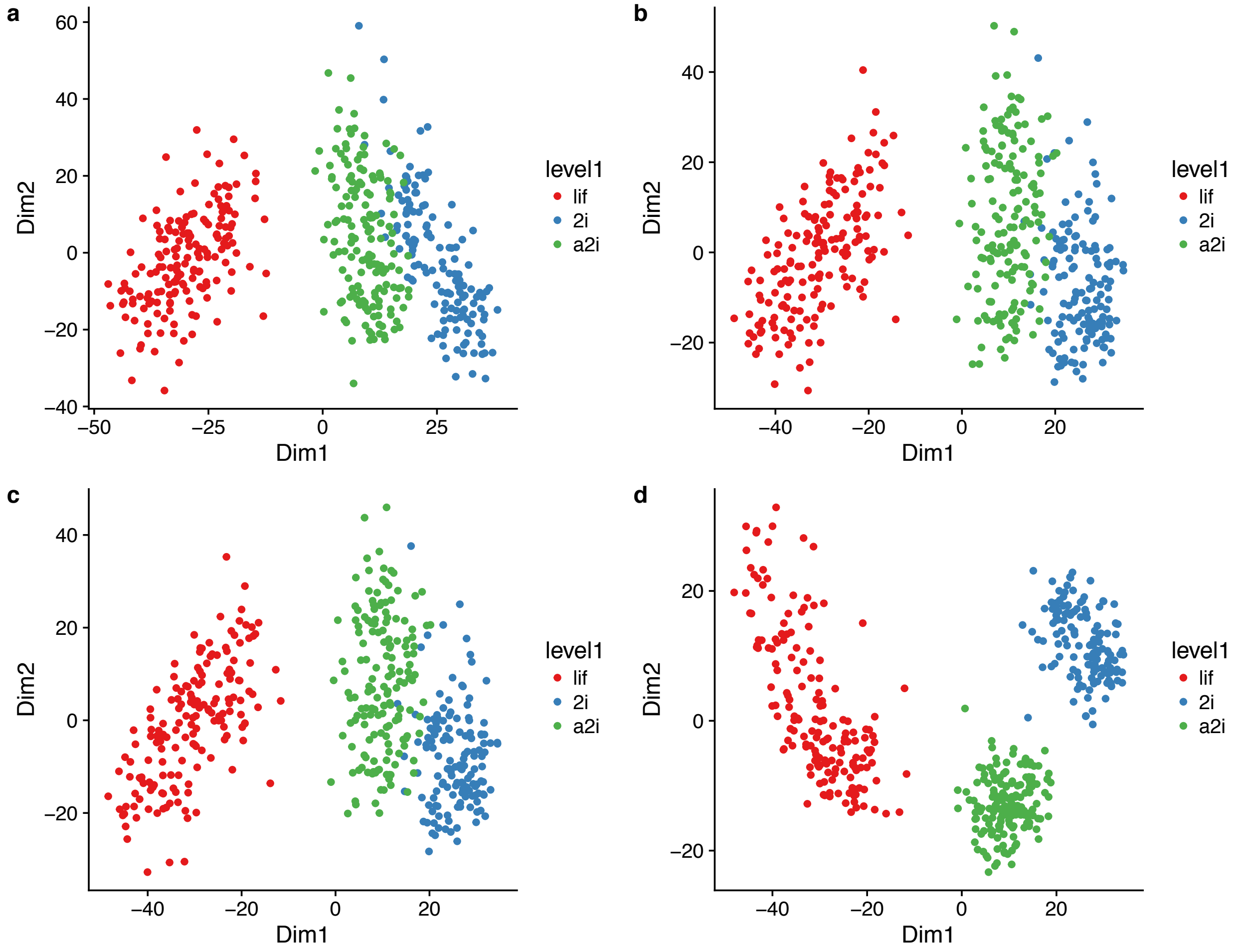

Supplementary Figure 12: *Principal component analysis for mESC dataset.* **(a)** No normalization; **(b)** TC normalization; **(c)** TMM normalization; **(d)** FQ normalization.

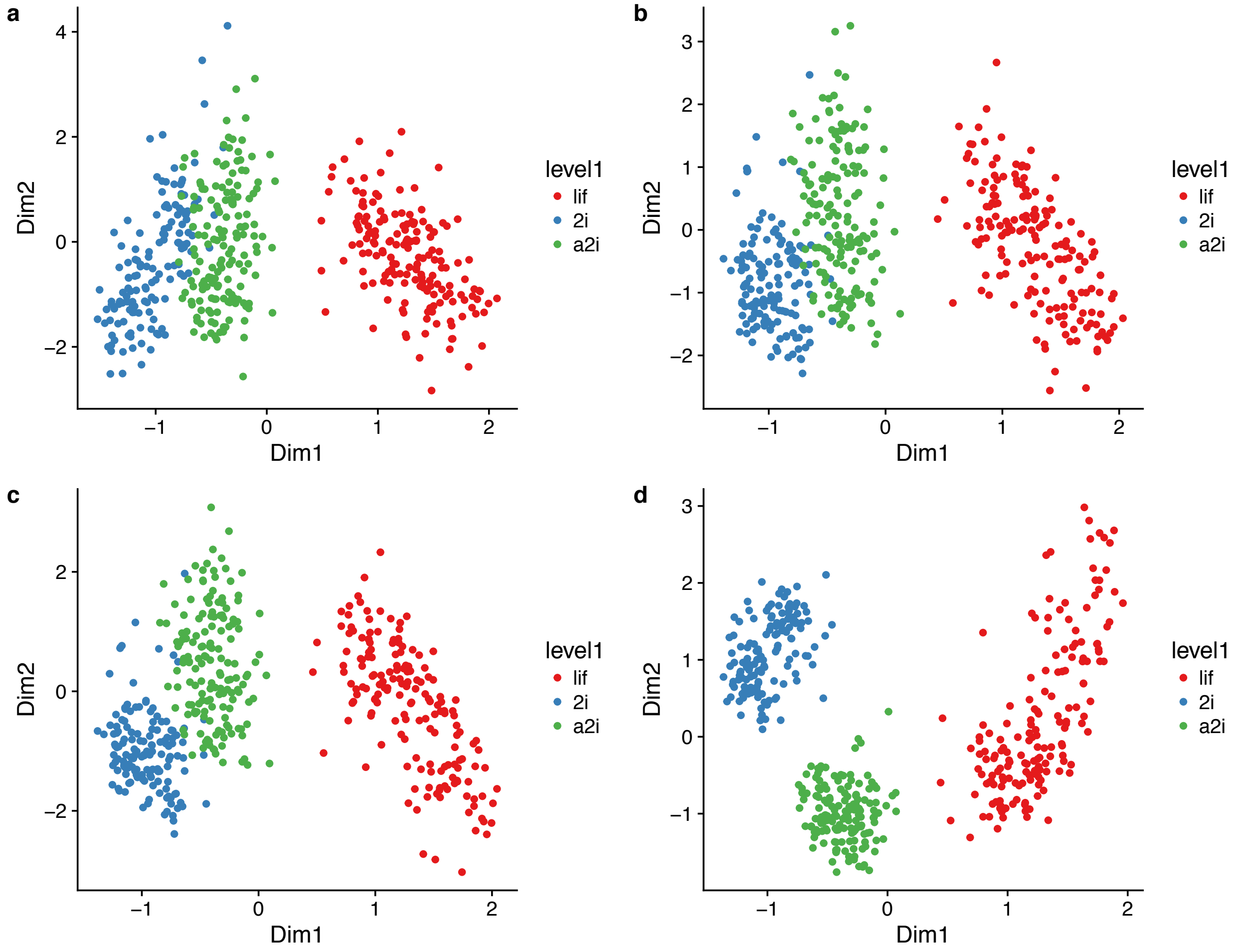

Supplementary Figure 13: *Zero-inflated factor analysis for mESC dataset.* **(a)** No normalization; **(b)** TC normalization; **(c)** TMM normalization; **(d)** FQ normalization.

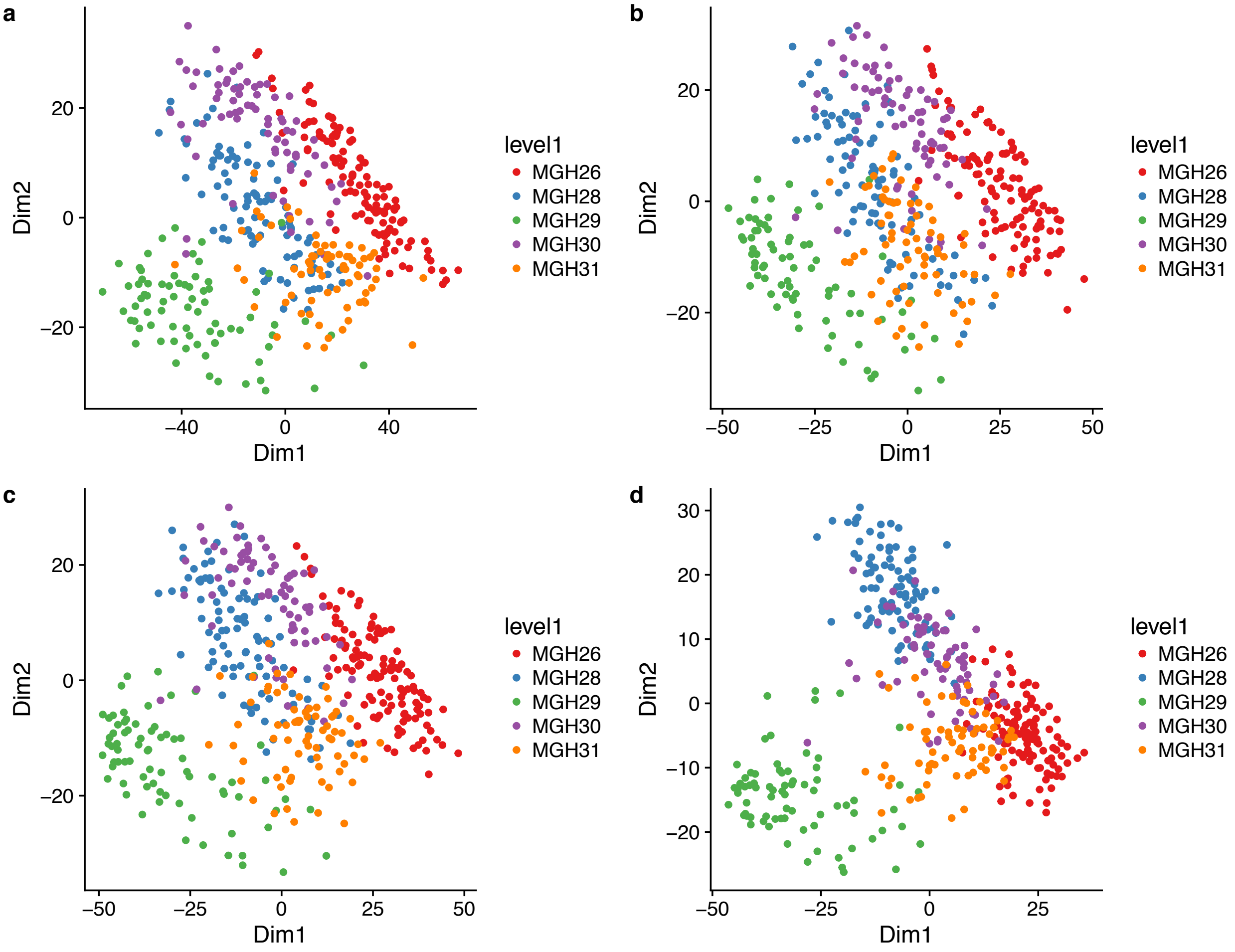

Supplementary Figure 14: *Principal component analysis for Glioblastoma dataset.* **(a)** No normalization; **(b)** TC normalization; **(c)** TMM normalization; **(d)** FQ normalization.

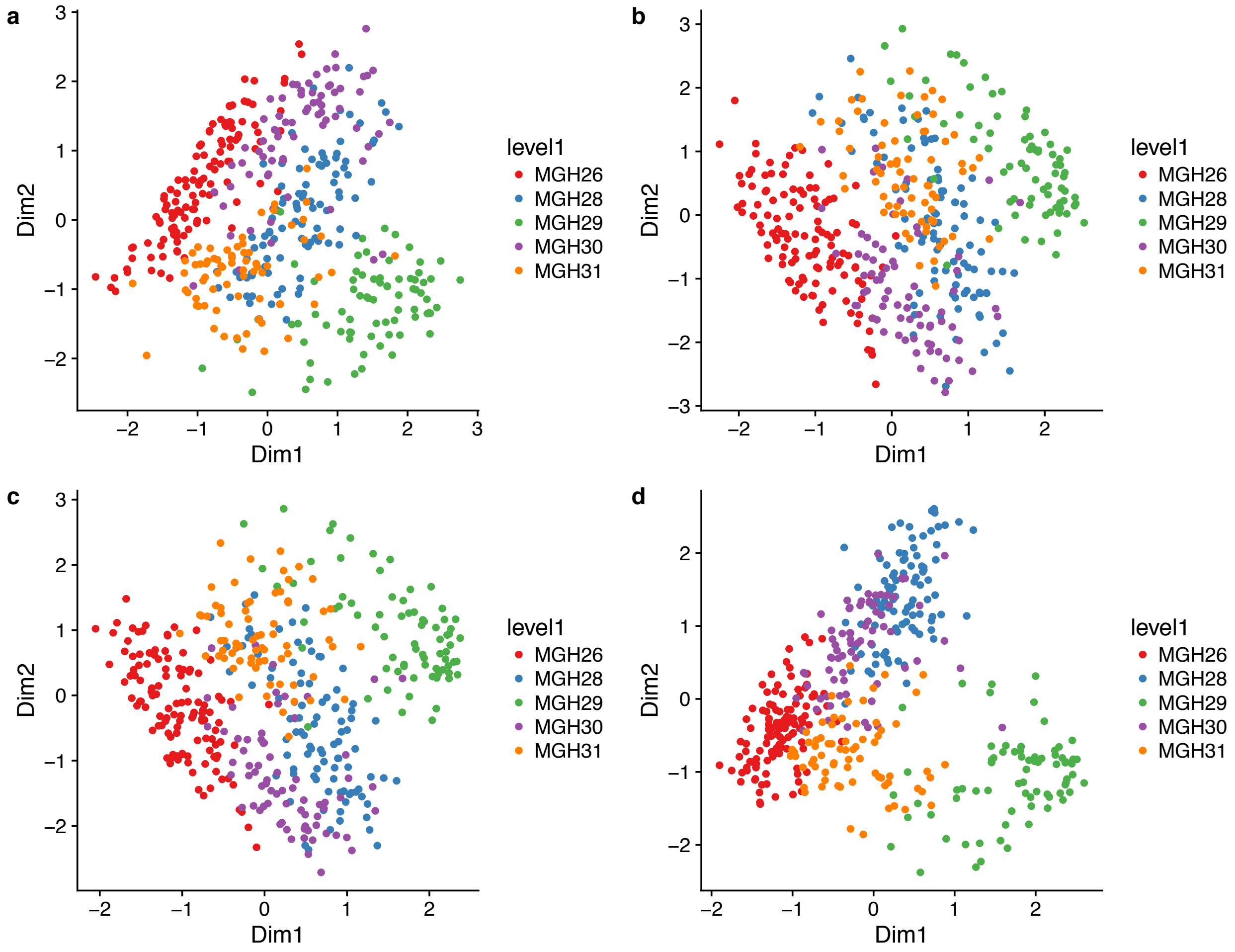

Supplementary Figure 15: *Zero-inflated factor analysis for Glioblastoma dataset.* **(a)** No normalization; **(b)** TC normalization; **(c)** TMM normalization; **(d)** FQ normalization.

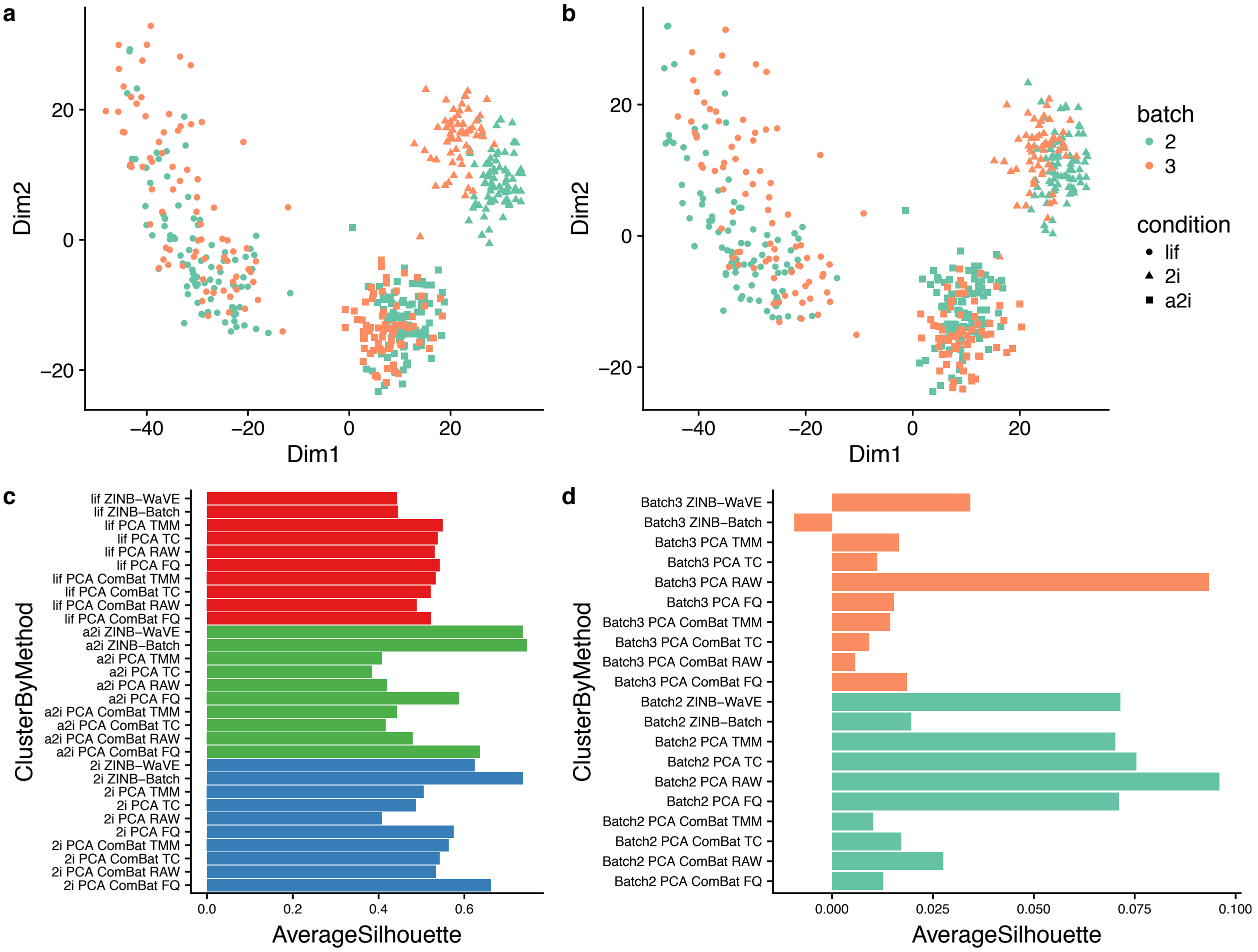

Supplementary Figure 16: *ZINB-Wave and ComBat: mESC dataset.* Upper panels provide two-dimensional representations of the data, with cells color-coded by batch and shape reflecting culture conditions: **(a)** PCA on FQ-normalized counts; **(b)** PCA on ComBat-normalized counts. **(c)** Average silhouette widths by biological condition for ZINB-WaVE with and without batch covariate, PCA with and without applying ComBat on raw counts and TC, TMM, and FQ-normalized counts; **(d)** Average silhouette widths by batch for ZINB-WaVE with and without batch covariate, PCA with and without applying ComBat on raw counts and TC, TMM, and FQ-normalized counts.

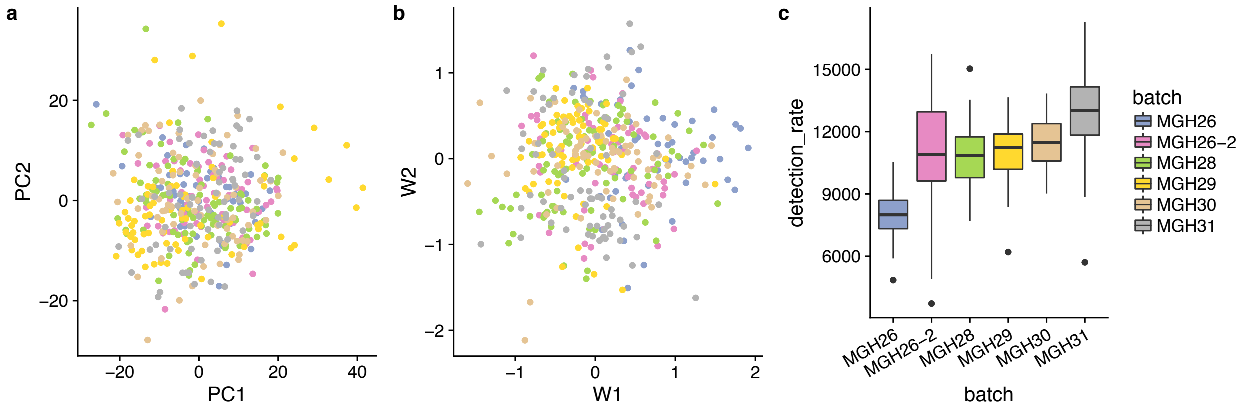

Supplementary Figure 17: *ZINB-Wave and ComBat: Glioblastoma dataset.* **(a)** PCA on FQ + ComBat-normalized counts; **(b)** ZINB-WaVE with batch as sample covariate; **(c)** Boxplot of sample detection rate stratified by batch. Sample detection rate is defined as the total number of genes with at least one read in a given sample.

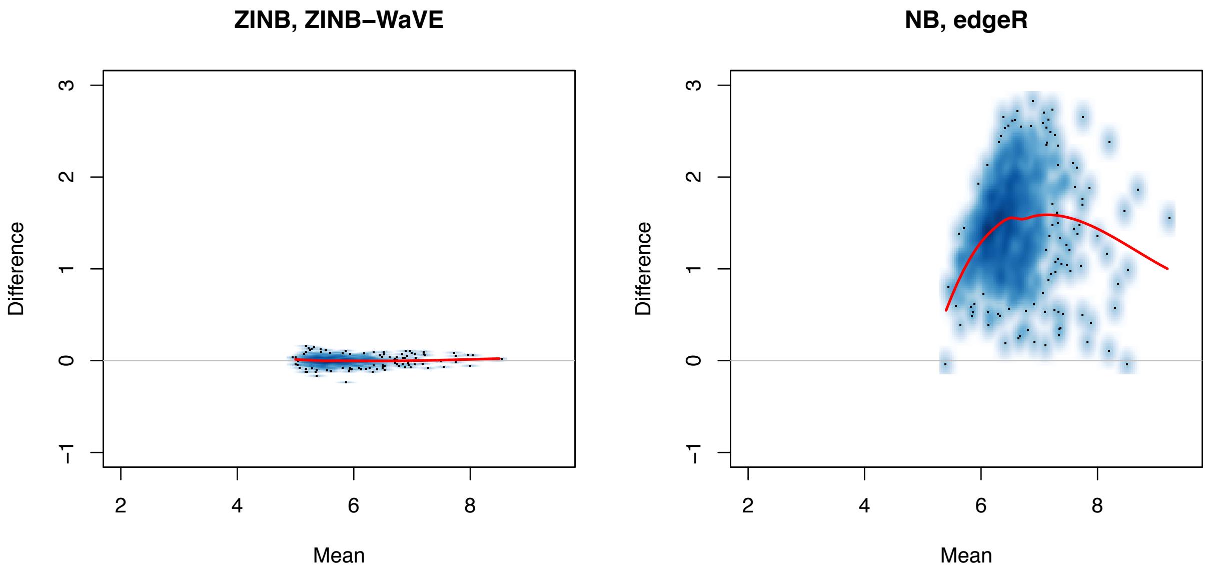

Supplementary Figure 18: *Goodness-of-fit of ZINB-WaVE and NB models: Mean-difference plots of estimated vs. observed mean count for V1 dataset.* Left panel: ZINB-WaVE. Right panel: Negative binomial model fit using edgeR package. Observed and estimated mean counts were averaged over n cells. Counts were plotted on a log scale. See Methods for details.

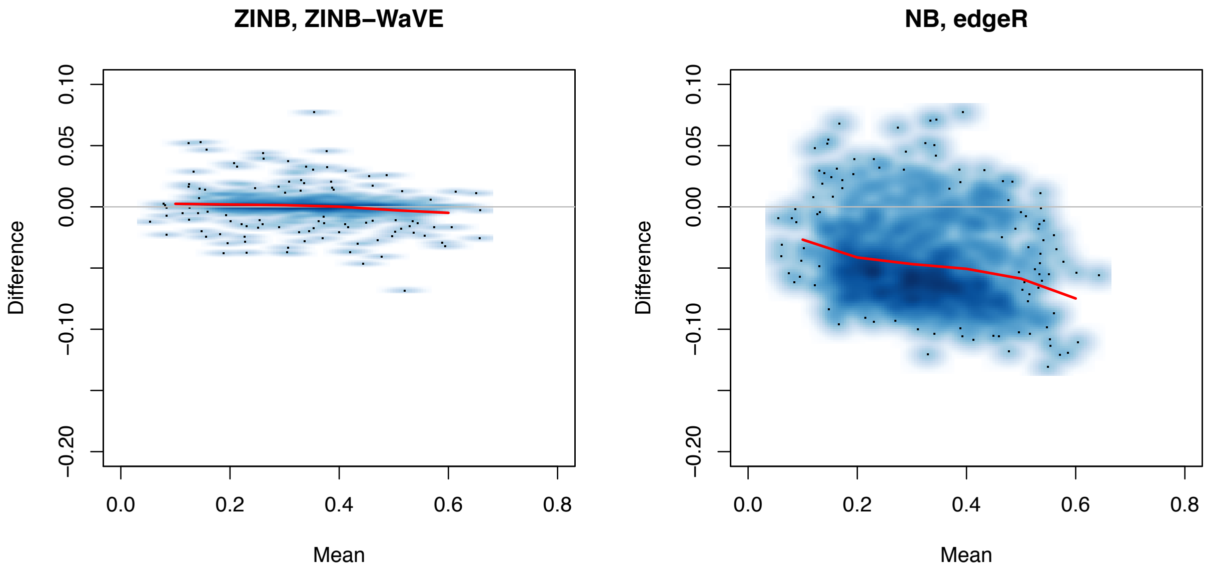

Supplementary Figure 19: *Goodness-of-fit of ZINB-WaVE and NB models: Mean-difference plots of estimated vs. observed zero probability for V1 dataset.* Left panel: ZINB-WaVE. Right panel: Negative binomial model fit using edgeR package. Observed and estimated zero probabilities were averaged over n cells. See Methods for details.

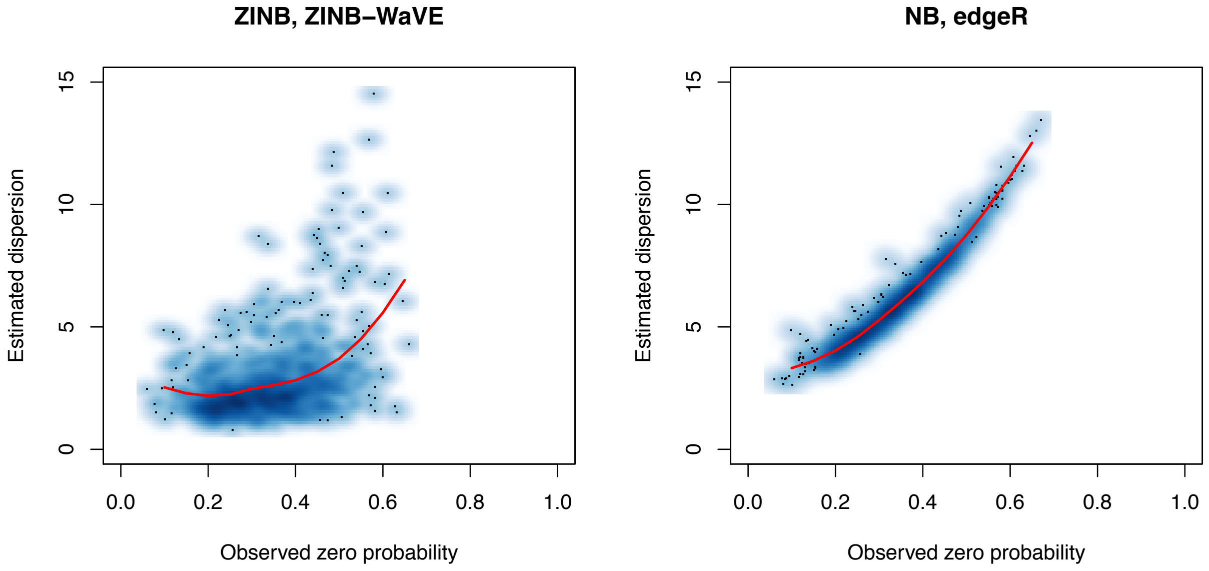

Supplementary Figure 20: *Goodness-of-fit of ZINB-WaVE and NB models: Estimated dispersion parameter vs. observed proportion of zero counts for V1 dataset.* Left panel: Genewise dispersion parameters ϕ_j estimated using ZINB-WaVE. Right panel: Genewise dispersion parameters ϕ_j estimated using edgeR package. See Methods for details.

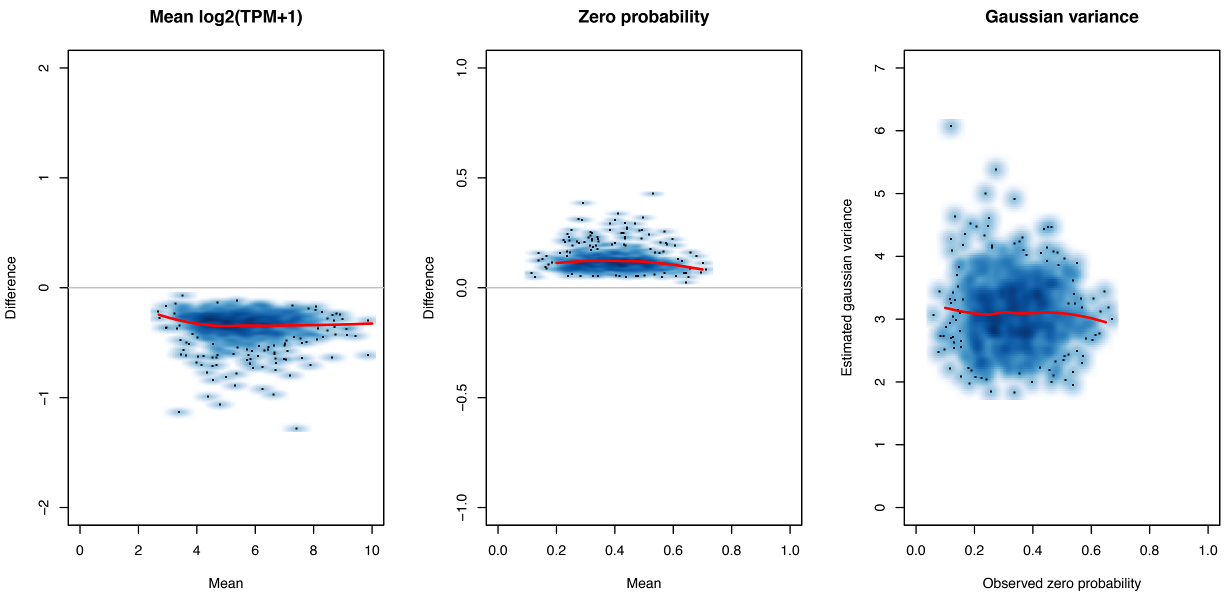

Supplementary Figure 21: *Goodness-of-fit of MAST hurdle model for V1 dataset.* Left panel: Mean-difference plot of estimated vs. observed mean log2(TPM+1). Middle panel: Mean-difference plot of estimated vs. observed zero probability. Right panel: Estimated Gaussian variance parameter σ_j^2 vs. observed proportion of zero counts. For left and middle panels, observed and estimated mean log2(TPM+1) and zero probabilities were averaged over n cells. Parameters were estimated using the function `zlm` from the MAST package, with an intercept and a covariate for the cellular detection rate (as recommended in the MAST vignette for the MAIT data analysis) for both the discrete and continuous parts.

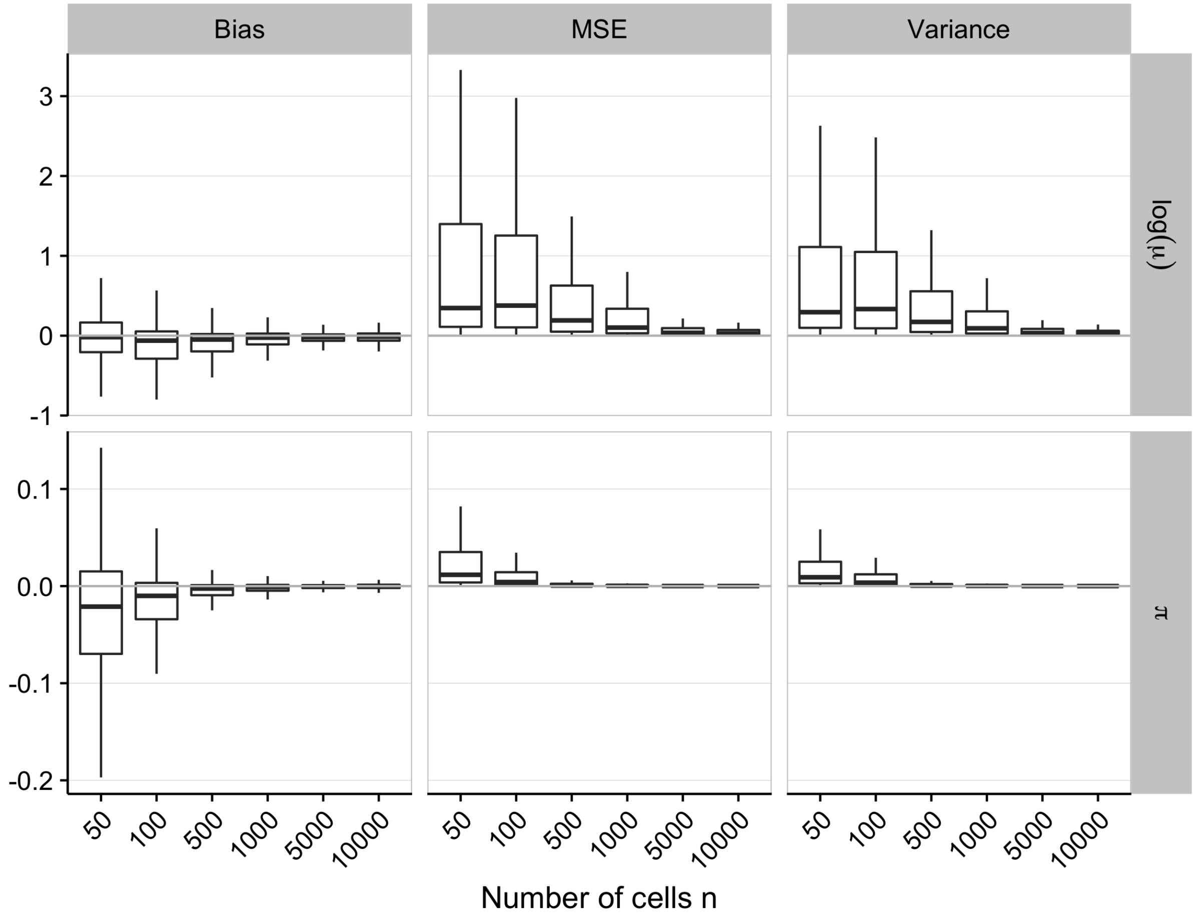

Supplementary Figure 22: *Bias, MSE, and variance for ZINB-WaVE estimation procedure: ZINB-WaVE simulation model.* Boxplots of bias, MSE, and variance for $\ln(\mu)$ and π as a function of the number of cells n. For each gene and cell, bias, MSE, and variance were averaged over $B = 10$ datasets simulated from our ZINB-WaVE model, based on the S1/CA1 dataset and with $n \in \{50, 100, 500, 1,000, 5,000, 10,000\}$ cells, $J = 1,000$ genes, scaling of one for the ratio of within to between-cluster sums of squares ($b^2 = 1$), and zero fraction of about 80%. The following values were used for both simulating the data and fitting the ZINB-WaVE model to these data: $K = 2$ unknown factors, $X = \mathbf{1}_n$, cell-level intercept ($V = \mathbf{1}_J$), and genewise dispersion.

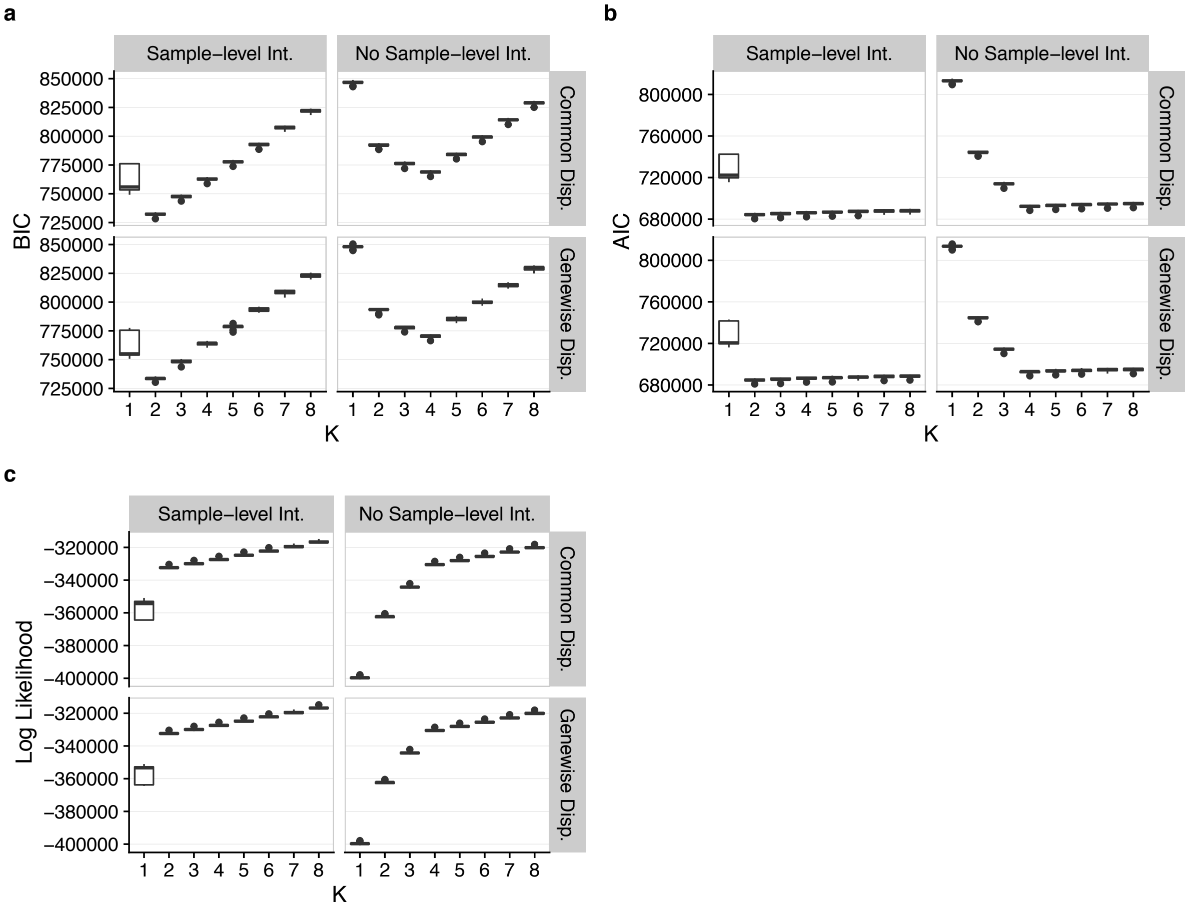

Supplementary Figure 23: *BIC, AIC, and log-likelihood for ZINB-WaVE estimation procedure: ZINB-WaVE simulation model.* Panels show boxplots of **(a)** BIC, **(b)** AIC, and **(c)** log-likelihood for ZINB-WaVE estimation procedure, as a function of the number of unknown covariates K. ZINB-WaVE was fit with $X = \mathbf{1}_n$, common/genewise dispersion, and with/without sample-level intercept (i.e., column of ones in gene-level covariate matrix V). For each gene and cell, BIC, AIC, and log-likelihood were averaged over $B = 10$ datasets simulated from our ZINB-WaVE model, based on the S1/CA1 dataset and with $n = 1,000$ cells, $J = 1,000$ genes, scaling of one for the ratio of within to between-cluster sums of squares ($b^2 = 1$), $K = 2$ unknown factors, zero fraction of about 80%, $X = \mathbf{1}_n$, cell-level intercept ($V = \mathbf{1}_J$), and genewise dispersion.

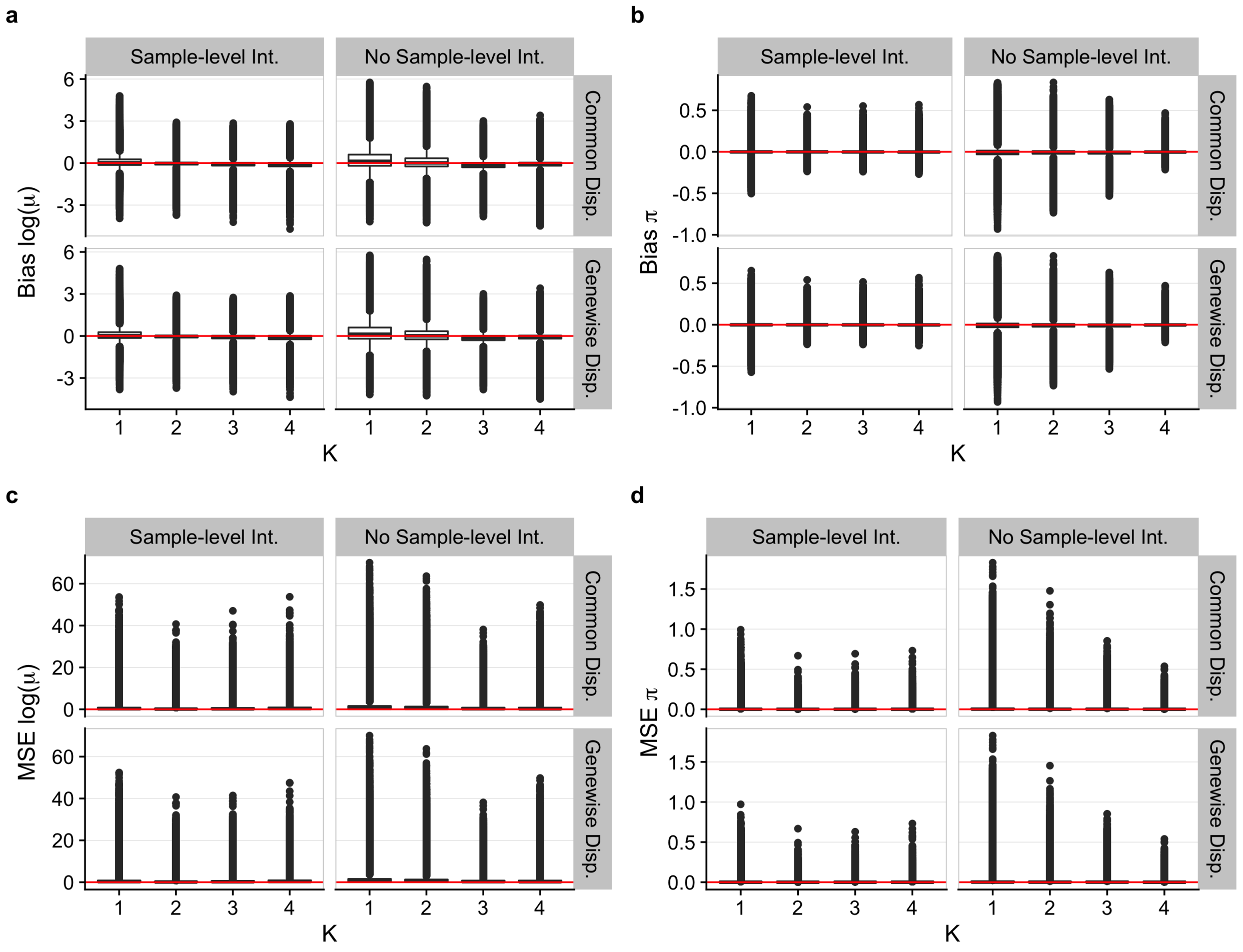

Supplementary Figure 24: *Bias and MSE for ZINB-WaVE estimation procedure: ZINB-WaVE simulation model.* Same as Figure 6, but with outliers plotted individually (i.e., observations beyond the whiskers).

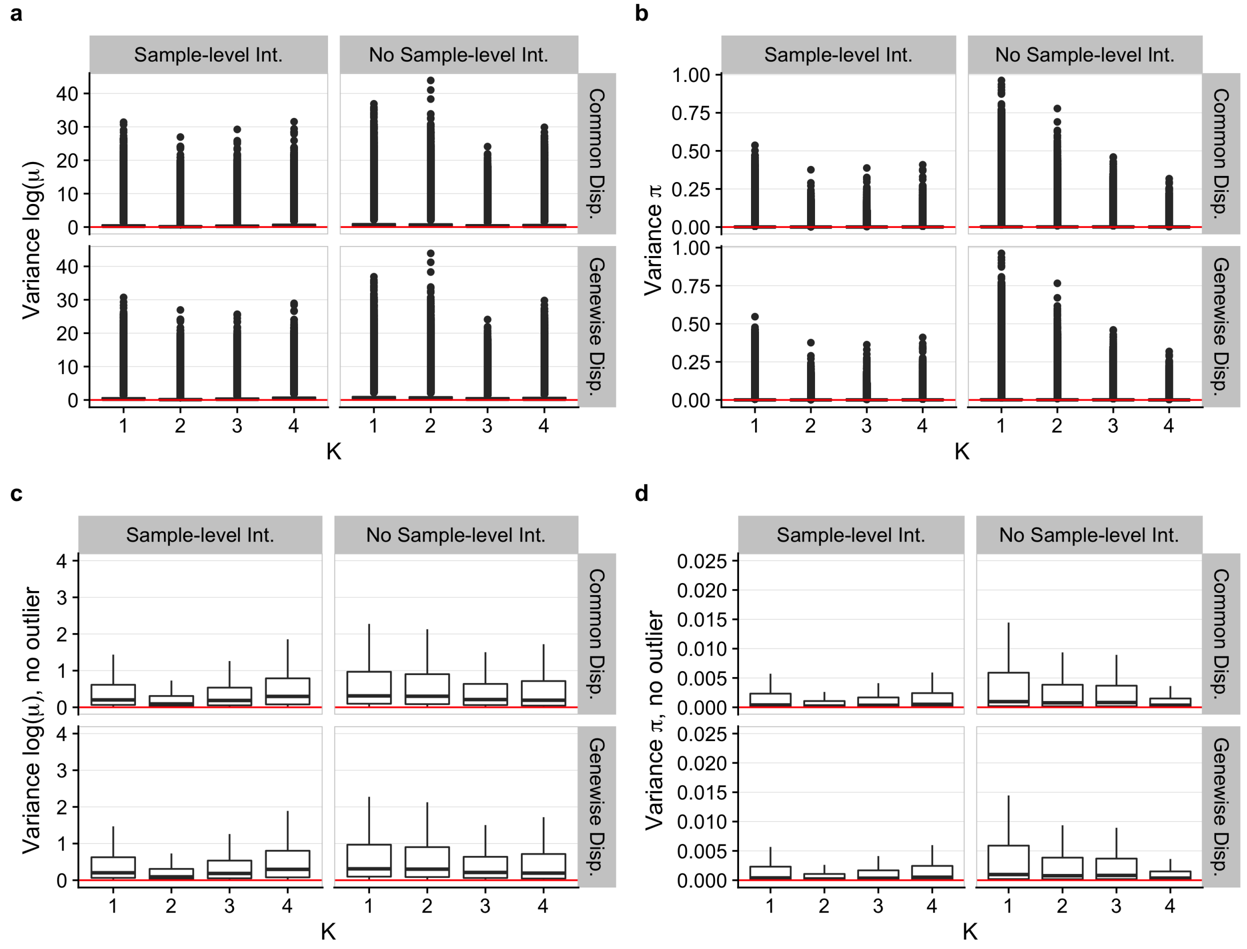

Supplementary Figure 25: *Variance for ZINB-WaVE estimation procedure: ZINB-WaVE simulation model.* Panels show boxplots of variance (over $B = 10$ simulated datasets) for estimates of $\ln(\mu)$ **(a, c)** and π **(b, d)**. Outliers plotted in **(a, b)** and omitted in **(c, d)**. Simulation scenario as in Figure 6.

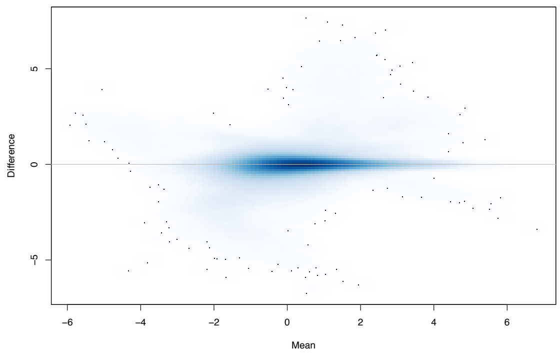

(a) $\ln(\mu)$

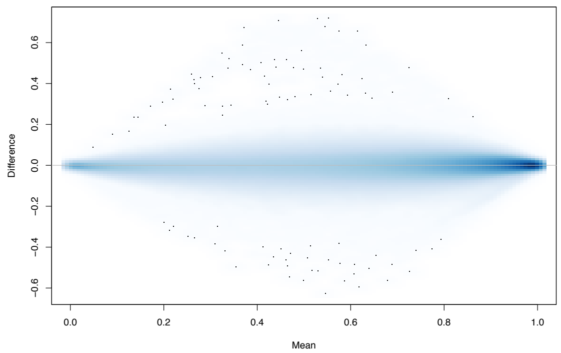

(b) π

Supplementary Figure 26: *Bias for ZINB-WaVE estimation procedure: ZINB-WaVE simulation model.* **(a)** Mean-difference plot of estimated vs. true negative binomial mean (log scale), $\ln(\hat{\mu}) - \ln(\mu)$ vs. $(\ln(\mu) + \ln(\hat{\mu}))/2$. **(b)** Mean-difference plot of estimated vs. true zero inflation probability, $\hat{\pi} - \pi$ vs. $(\pi + \hat{\pi})/2$. The estimates are based on one of the $B = 10$ datasets simulated from our ZINB-WaVE model, based on the S1/CA1 dataset and with $n = 1,000$ cells, $J = 1,000$ genes, scaling of one for the ratio of within to between-cluster sums of squares ($b^2 = 1$), and zero fraction of about 80%. The following values were used for both simulating the data and fitting the ZINB-WaVE model to these data: $K = 2$ unknown factors, $X = \mathbf{1}_n$, cell-level intercept ($V = \mathbf{1}_J$), and genewise dispersion (as in Fig. 6 and Supplementary Fig. 24 and 25).

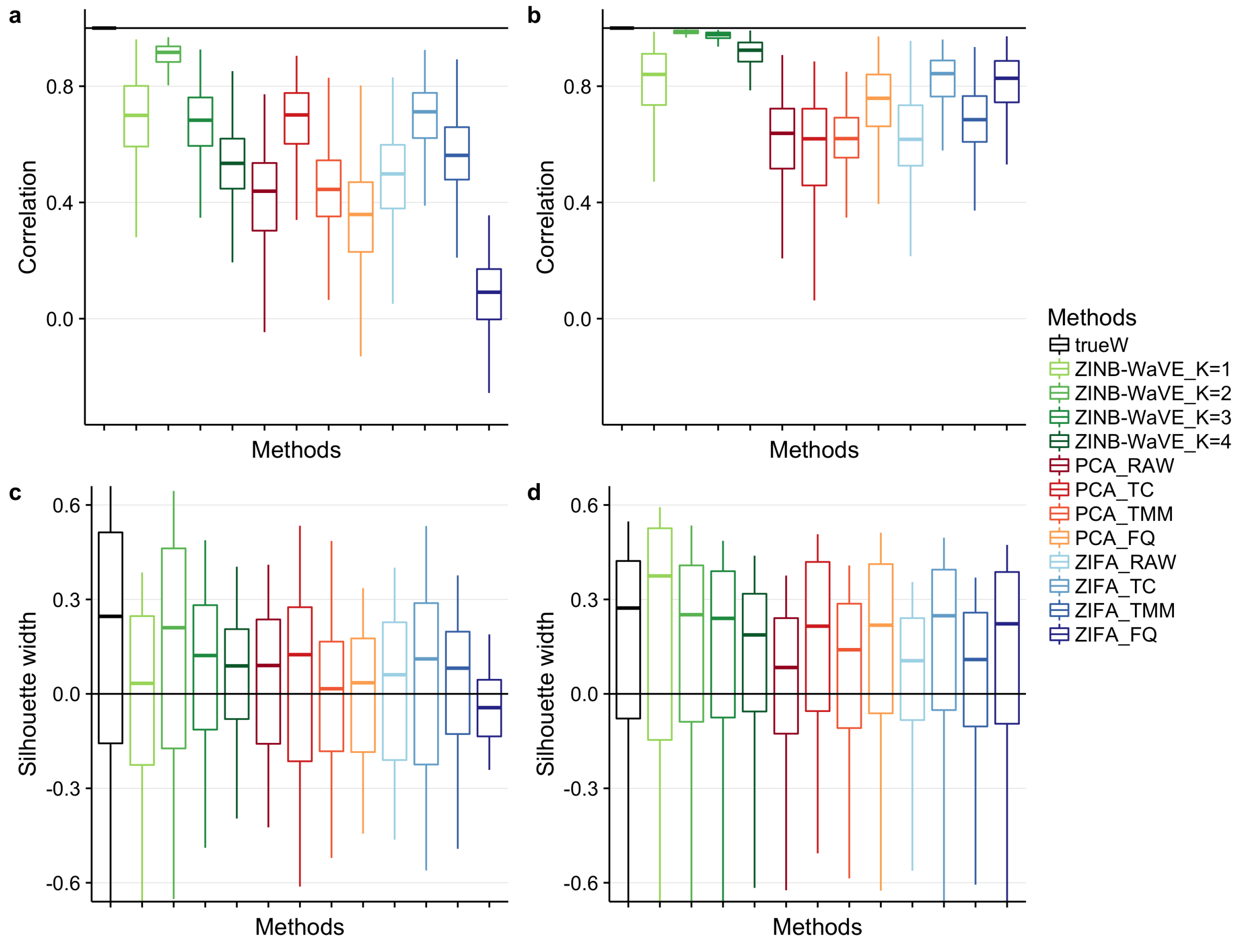

Supplementary Figure 27: *Between-sample distances and silhouette widths for ZINB-WaVE, PCA, and ZIFA: ZINB-WaVE simulation model.* **(a)** Boxplots of correlations between between-sample distances based on true and estimated low-dimensional representations of the data for simulations based on the V1 dataset. **(b)** Same as **(a)** for simulations based on the S1/CA1 dataset. **(c)** Boxplots of silhouette widths for true clusters for simulations based on the V1 dataset. **(d)** Same as **(c)** for simulations based on the S1/CA1 dataset. All datasets were simulated from our ZINB-WaVE model with $n = 10,000$ cells, $J = 1,000$ genes, "harder" clustering ($b^2 = 5$), $K = 2$ unknown factors, zero fraction of about 80%, $X = \mathbf{1}_n$, cell-level intercept ($V = \mathbf{1}_J$), and genewise dispersion. Each boxplot is based on n values corresponding to each of the n samples and defined as averages of correlations **(a, b)** or silhouette widths **(c, d)** over $B = 10$ simulations. Between-sample distance matrices and silhouette widths were based on W for ZINB-WaVE, the first two principal components for PCA, and the first two latent variables for ZIFA. ZINB-WaVE was applied with $X = \mathbf{1}_n$, $V = \mathbf{1}_J$, genewise dispersion, and $K \in \{1, 2, 3, 4\}$. For PCA and ZIFA, different normalization methods were used. Colors correspond to the different methods. See Figure 7a–d for the same scenario but with $n = 1,000$ cells and Supplementary Figure 28 for additional scenarios.

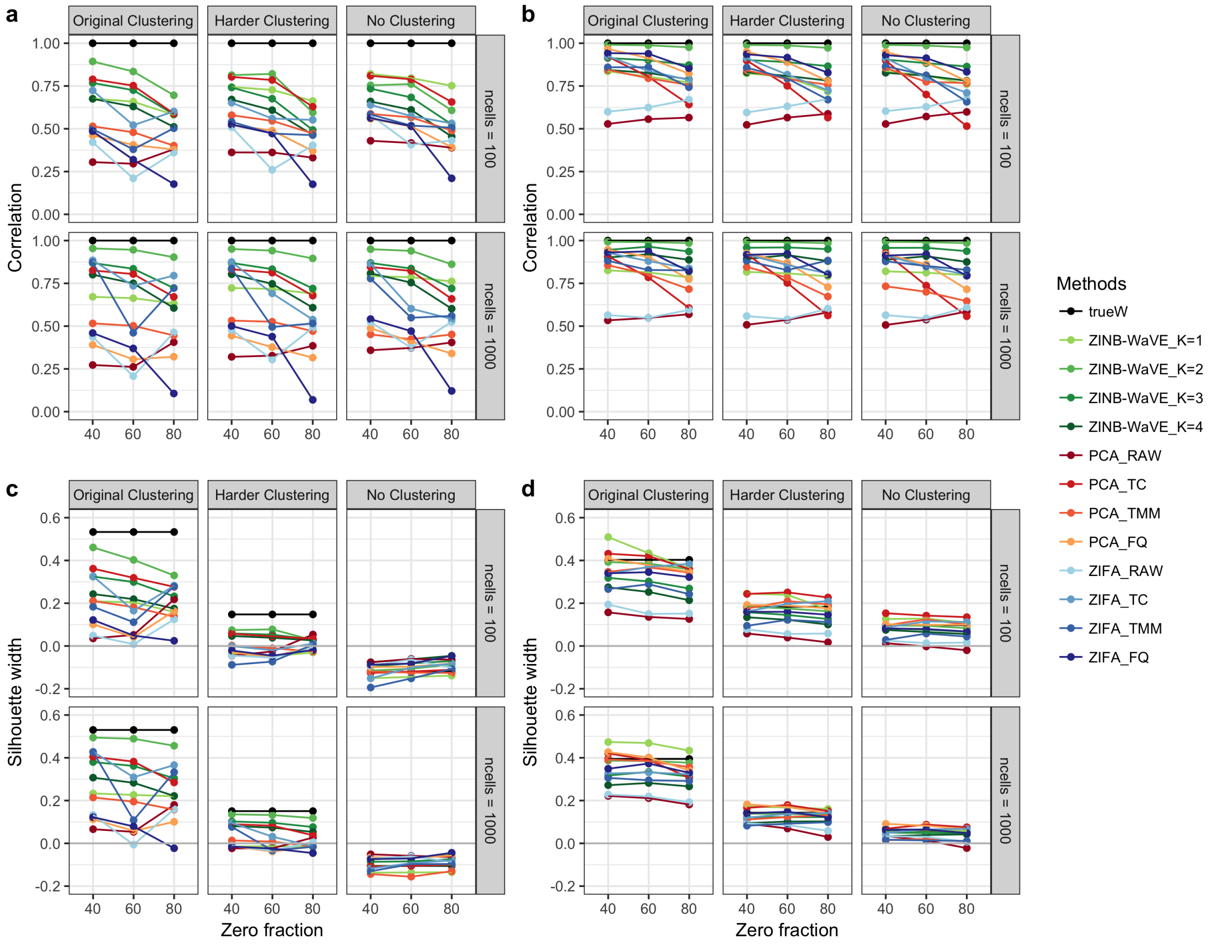

Supplementary Figure 28: *Between-sample distances and silhouette widths for ZINB-WaVE, PCA, and ZIFA: ZINB-WaVE simulation model.* **(a)** Correlation between between-sample distances based on true and estimated low-dimensional representations of the data for simulations based on the V1 dataset. **(b)** Same as **(a)** for simulations based on the S1/CA1 dataset. **(c)** Silhouette width for true clusters for simulations based on the V1 dataset. **(d)** Same as **(c)** for simulations based on the S1/CA1 dataset. As in Figure 7, all datasets were simulated from our ZINB-WaVE model with $J = 1,000$ genes, $K = 2$ unknown factors, $X = \mathbf{1}_n$, cell-level intercept ($V = \mathbf{1}_J$), and genewise dispersion. Each point corresponds to a simulation scenario (zero fraction, clustering strength, sample size); correlations between true and estimated between-sample distances and silhouette widths are averaged over $B = 10$ simulated datasets and n cells. Column panels show three different clustering scenarios, where the scaling of the ratio of within to between-cluster sums of squares b^2 corresponds to the original clustering ($b^2 = 1$), a harder clustering ($b^2 = 5$), and no clustering ($b^2 = 10$). Row panels correspond to different numbers of cells ($n \in \{100, 1,000\}$). Between-sample distance matrices and silhouette widths were based on W for ZINB-WaVE, the first two principal components for PCA, and the first two latent variables for ZIFA. ZINB-WaVE was applied with $X = \mathbf{1}_n$, $V = \mathbf{1}_J$, genewise dispersion, and $K \in \{1, 2, 3, 4\}$. For PCA and ZIFA, different normalization methods were used. Colors correspond to the different methods.

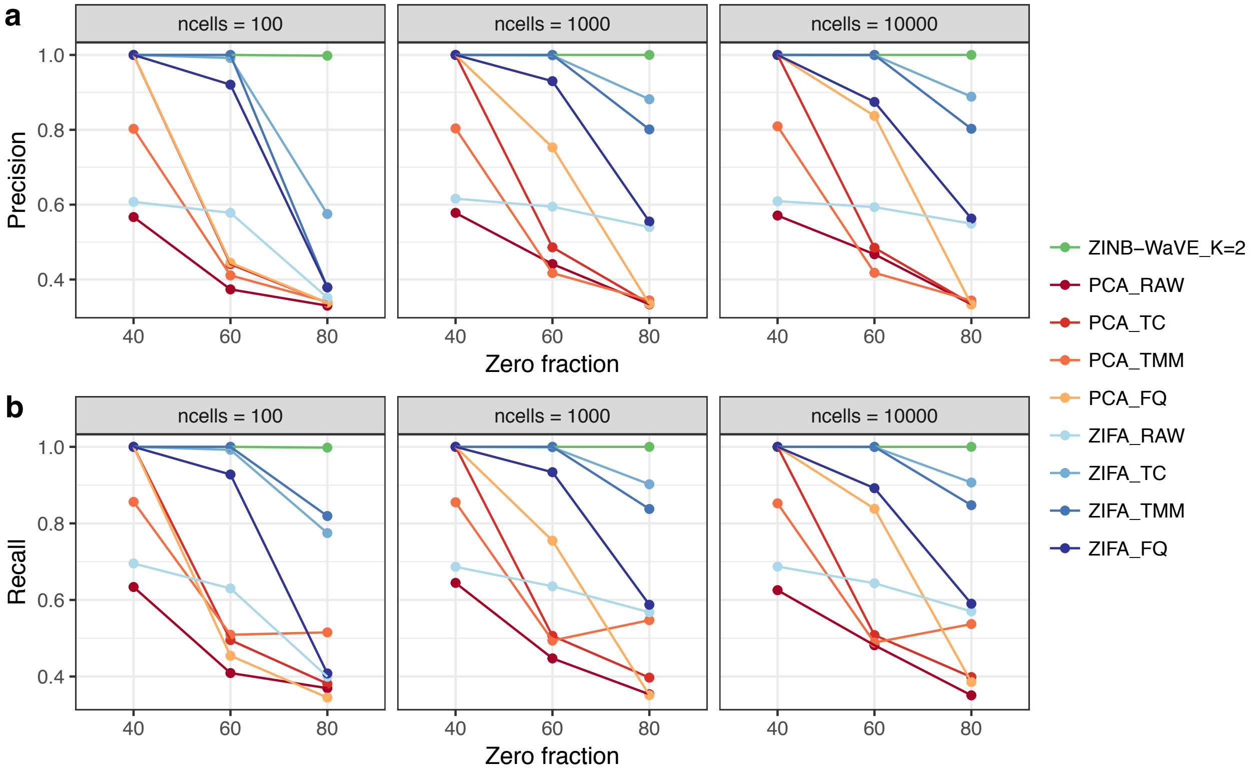

Supplementary Figure 29: *Precision and recall for ZINB-WaVE, PCA, and ZIFA: Lun & Marioni*[42] *simulation model.* Average **(a)** precision coefficient and **(b)** recall coefficient (over n samples and $B = 10$ simulations) vs. zero fraction, for $n \in \{100, 1,000, 10,000\}$ cells, for datasets simulated from the Lun & Marioni[42] model, with $C = 3$ clusters and equal number of cells per cluster. Clustering was performed using k-means on W for ZINB-WaVE, the first two principal components for PCA, and the first two latent variables for ZIFA. ZINB-WaVE was applied with $X = \mathbf{1}_n$, $V = \mathbf{1}_J$, genewise dispersion, and $K = 2$. For PCA and ZIFA, different normalization methods were used. Colors correspond to the different methods. While ZINB-WaVE has a recall and precision of one for all sample sizes n and zero fractions, the performance of PCA and ZIFA decreases with larger zero fraction. See Methods for details on clustering procedure and precision and recall coefficients.

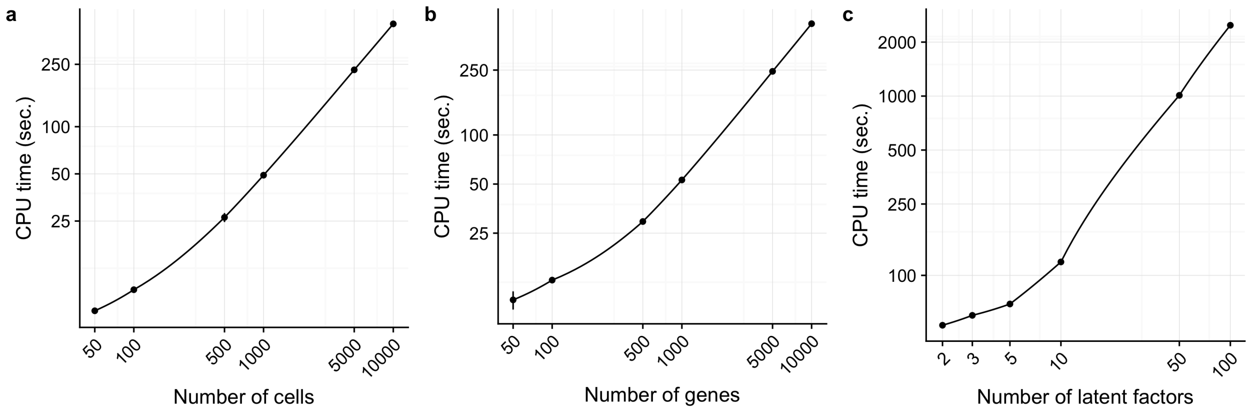

Supplementary Figure 30: *CPU time for ZINB-WaVE estimation procedure.* Log-log scatterplot of mean CPU time (in seconds) vs. **(a)** sample size n, **(b)** number of genes J, and **(c)** number of latent factors K. For each panel $B = 10$ datasets were simulated from the Lun & Marioni[42] model with zero fraction of about 60%. The following specific values were used for each panel: **(a)** $n \in \{50, 100, 500, 1,000, 5,000, 10,000\}$ cells, $J = 1,000$ genes, $K = 2$ latent factors; **(b)** $J \in \{50, 100, 500, 1,000, 5,000, 10,000\}$ genes, $n = 1,000$ cells, $K = 2$ latent factors; **(c)** $n = 1,000$ cells, $J = 1,000$ genes, and $K \in \{2, 3, 5, 10, 50, 100\}$ latent factors. The following values were used to fit the ZINB-WaVE model: $X = \mathbf{1}_n$, cell-level intercept ($V = \mathbf{1}_J$), and common dispersion. CPU times were averaged over $B = 10$ simulated datasets and standard deviations are indicated by the vertical bars. Computations were done with 7 cores on an iMac with eight 4 GHz Intel Core i7 CPUs and 32 GB of RAM.
